# Functional divergence of a global regulatory complex governing fungal filamentation

**DOI:** 10.1371/journal.pgen.1007901

**Published:** 2019-01-07

**Authors:** Elizabeth J. Polvi, Amanda O. Veri, Zhongle Liu, Saif Hossain, Sabrina Hyde, Sang Hu Kim, Faiza Tebbji, Adnane Sellam, Robert T. Todd, Jinglin L. Xie, Zhen-Yuan Lin, Cassandra J. Wong, Rebecca S. Shapiro, Malcolm Whiteway, Nicole Robbins, Anne-Claude Gingras, Anna Selmecki, Leah E. Cowen

**Affiliations:** 1 Department of Molecular Genetics, University of Toronto, Ontario, Canada; 2 Infectious Disease Research Centre, Université Laval, Quebec, Canada; 3 Department of Medical Microbiology and Immunology, Creighton University School of Medicine, Omaha, Nebraska, United States of America; 4 Lunenfeld-Tanenbaum Research Institute, Mount Sinai Hospital, Ontario, Canada; 5 Department of Biology, Concordia University, Quebec, Canada; Carnegie Mellon University, UNITED STATES

## Abstract

Morphogenetic transitions are prevalent in the fungal kingdom. For a leading human fungal pathogen, *Candida albicans*, the capacity to transition between yeast and filaments is key for virulence. For the model yeast *Saccharomyces cerevisiae*, filamentation enables nutrient acquisition. A recent functional genomic screen in *S*. *cerevisiae* identified Mfg1 as a regulator of morphogenesis that acts in complex with Flo8 and Mss11 to mediate transcriptional responses crucial for filamentation. In *C*. *albicans*, Mfg1 also interacts physically with Flo8 and Mss11 and is critical for filamentation in response to diverse cues, but the mechanisms through which it regulates morphogenesis remained elusive. Here, we explored the consequences of perturbation of Mfg1, Flo8, and Mss11 on *C*. *albicans* morphogenesis, and identified functional divergence of complex members. We observed that *C*. *albicans* Mss11 was dispensable for filamentation, and that overexpression of *FLO8* caused constitutive filamentation even in the absence of Mfg1. Harnessing transcriptional profiling and chromatin immunoprecipitation coupled to microarray analysis, we identified divergence between transcriptional targets of Flo8 and Mfg1 in *C*. *albicans*. We also established that Flo8 and Mfg1 cooperatively bind to promoters of key regulators of filamentation, including *TEC1*, for which overexpression was sufficient to restore filamentation in the absence of Flo8 or Mfg1. To further explore the circuitry through which Mfg1 regulates morphogenesis, we employed a novel strategy to select for mutations that restore filamentation in the absence of Mfg1. Whole genome sequencing of filamentation-competent mutants revealed chromosome 6 amplification as a conserved adaptive mechanism. A key determinant of the chromosome 6 amplification is *FLO8*, as deletion of one allele blocked morphogenesis, and chromosome 6 was not amplified in evolved lineages for which *FLO8* was re-located to a different chromosome. Thus, this work highlights rewiring of key morphogenetic regulators over evolutionary time and aneuploidy as an adaptive mechanism driving fungal morphogenesis.

## Author summary

Fungal infections pose a severe burden to human health worldwide. *Candida albicans* is a leading cause of systemic fungal infections, with mortality rates approaching 40%. One of the key virulence traits of this fungus is its ability to transition between yeast and filamentous forms in response to diverse host-relevant cues. The model yeast *Saccharomyces cerevisiae* is also capable of filamentous growth in certain conditions, and previous work has identified a key transcriptional complex required for filamentation in both species. However, here we discover that the circuitry governed by this complex in *C*. *albicans* is largely distinct from that in the non-pathogenic *S*. *cerevisiae*. We also employ a novel selection strategy to perform experimental evolution, identifying chromosome triplication as a mechanism to restore filamentation in a non-filamentous mutant. This work reveals unique circuitry governing a key virulence trait in a leading fungal pathogen, identifying potential therapeutic targets to combat these life-threatening infections.

## Introduction

The fungal kingdom is recognized for its vast morphological plasticity, with many species capable of undergoing morphogenetic transformations in response to diverse environmental cues. For instance, filamentous fungi such as *Aspergillus fumigatus* undergo spore germination and branching throughout their life cycle, dermatophyte fungal pathogens produce arthroconidia, and thermally dimorphic fungi such as *Histoplasma capsulatum* exist as filamentous mycelia at ambient temperatures and transition to a yeast form upon exposure to mammalian physiological temperatures [1–3]. The purposes of such transformations are equally diverse, with morphogenesis playing critical roles in sexual reproduction, nutrient acquisition and virulence. For example, the basidiomycete *Cryptococcus neoformans* forms elongated filaments for the purpose of mating or monokaryotic fruiting [4], and the model yeast *Saccharomyces cerevisiae* undergoes invasive or pseudohyphal growth for nutrient acquisition under starvation conditions [5]. Finally, the fungal pathogen *Candida albicans* transitions from a yeast to hyphal state in response to a variety of host-relevant cues [2,6], which aids in tissue invasion, immune cell evasion, and biofilm formation, such that morphogenesis is critical for virulence of this pathogen [2,7,8]. Thus, fungal morphogenesis comprises a diversity of processes required for cellular survival, proliferation, and pathogenesis.

The past several decades have witnessed a surge in the frequency of life-threatening human fungal infections, making mycotic disease a serious public health problem. *Candida* species comprise one of the leading genera of human fungal pathogens, with *C*. *albicans* being the most prevalent [9]. Those most susceptible to these infections are the increasing population of immunocompromised patients, including those undergoing chemotherapy or transplantation, as well as those infected with HIV. *C*. *albicans* can cause fatal bloodstream infections, with mortality rates approaching 40% despite therapeutic intervention [9,10]. This is in part due to a limited number of antifungals available to treat systemic infections coupled with the frequent emergence of antifungal drug resistance in the clinic [11]. Further, the capacity of *C*. *albicans* to cause life-threatening disease in its human host is enabled by a complex repertoire of virulence factors, including the expression of surface structures that mediate adherence to epithelial cells, the secretion of hydrolytic enzymes that induce host cell damage, the capacity to produce biofilms that are intrinsically resistant to antifungal drugs, and the ability to transition between yeast and filamentous growth states [2,12–14]. Given the dearth of new antifungal classes uncovered by traditional approaches of targeting essential proteins required for viability, a complementary approach of targeting proteins required for pathogen virulence may prove to be a useful strategy to combat fungal infections [15].

The ability of *C*. *albicans* to transition between yeast and filamentous forms occurs upon exposure to a variety of different cues, including host febrile temperature, serum, and nutrient depletion [2,6]. Complex genetic circuitry underpins this transition, and distinct mechanisms are involved in initiating and maintaining filamentous growth [8,16–19]. One of the core pathways required for filamentation in *C*. *albicans* is the cAMP-protein kinase A (PKA) signaling cascade, for which multiple components of the pathway are required for filamentous growth in response to diverse conditions [2,6]. In *C*. *albicans*, the PKA complex is composed of the regulatory subunit Bcy1 and two catalytic subunits, Tpk1 and Tpk2 [20]. Increased cAMP levels produced by the adenylyl cyclase Cyr1 activate the PKA complex, resulting in phosphorylation and activation of the transcription factor Efg1, which governs the expression of many hyphal-specific genes [6,21,22]. Other signaling pathways including the Cek1-mitogen activated protein kinase (MAPK) pathway [23], Rim101 pH sensing pathway [24], and protein kinase C (Pkc1) cascade [25] also play important roles in enabling filamentation in response to diverse stimuli. Despite the continued identification and characterization of genes involved in *C*. *albicans* morphogenesis, an understanding of the regulatory networks governing morphogenesis remains largely elusive.

Systematic analyses of genes enabling filamentous growth have been performed on a genomic level in both *S*. *cerevisiae* and *C*. *albicans* [18,26]. Such analyses have revealed a striking divergence in the sets of genes required for filamentation between these species [18,26]. A recent study with the *S*. *cerevisiae* Σ1278b strain defined genes required for: a) diploid pseudohyphal formation in response to low nitrogen; b) haploid invasive growth in response to glucose depletion; and c) biofilm formation on semi-solid agar [26]. Despite largely distinct gene sets being important for each of these filamentation programs, there was a core gene set required for morphogenesis, including the previously uncharacterized protein Mfg1 [26]. In *S*. *cerevisiae*, Mfg1 forms a complex with the transcriptional regulators Flo8 and Mss11 to control the expression of hundreds of genes, including *FLO11*, which encodes a cell surface glycoprotein essential for filamentation [26,27]. Members of the *S*. *cerevisiae* Flo8-Mfg1-Mss11 complex primarily function in concert to promote morphogenesis. Of the 152 promoters bound by *S*. *cerevisiae* Mfg1, 89% are also bound by Flo8 and 78% are bound by Mss11 [26]. Further, deletion of *S*. *cerevisiae MFG1* reduces Flo8 binding to 90.8% of its target promoters [26]. Mfg1 is also a key regulator of morphogenesis in *C*. *albicans*, and the physical interaction with Flo8 and Mss11 is conserved under basal conditions [26]. However, the mechanisms through which this complex regulates filamentous growth in *C*. *albicans* remains largely enigmatic given that the transcriptional targets of this complex remain unknown and that no *FLO11* homolog has been identified in this fungal pathogen.

In this study, we characterized the role of the Flo8-Mfg1-Mss11 complex in regulating *C*. *albicans* filamentation. Although Flo8 and Mfg1 were required for filamentous growth in response to numerous conditions, they were not required for morphogenesis induced by compromised function of the molecular chaperone Hsp90, demonstrating that these mutants are capable of filamentation in response to specific cues. Further, overexpression of *FLO8* resulted in constitutive hyphal growth yet overexpression of *MFG1* did not, suggesting distinct functions of these regulators in the yeast-to-hyphal transition. We observed functional divergence of the complex members, as Mss11 was dispensable for filamentous growth and had a reduced physical interaction with Mfg1 and Flo8 in filament-inducing cues. Surprisingly, *MSS11* overexpression was sufficient to induce morphogenesis in the absence of an inducing cue, highlighting the complex functional relationships. Chromatin immunoprecipitation (ChIP) of TAP-tagged Flo8 or Mfg1 followed by microarray analysis (ChIP-chip) revealed significant divergence between the transcriptional targets of Flo8 and Mfg1 in *C*. *albicans*, and highlighted dynamic temporal changes in promoter occupancy and gene expression in response to serum as a filament-inducing cue. However, Mfg1 and Flo8 cooperatively bound to a subset of key targets involved in morphogenesis, including *TEC1*, for which overexpression is sufficient to drive filamentation in the absence of either Mfg1 or Flo8. Finally, to gain further mechanistic insight into the circuitry through which Mfg1 regulates morphogenesis, we employed a novel experimental evolution strategy based on introduction of a drug resistance marker under the control of a filament-specific promoter to select for *mfg1Δ/mfg1Δ* mutants with a restored ability to filament in response to serum. Whole genome sequencing of mutants capable of filamentation in the absence of Mfg1 uncovered an adaptive mechanism involving amplification of chromosome 6, on which *FLO8* is located. Thus, we highlight Mfg1 as a critical regulator of *C*. *albicans* morphogenesis, uncover distinct roles of Flo8, Mfg1, and Mss11 in promoting filamentation, and provide a striking example of aneuploidy formation as a mechanism of adaptive evolution to enable filamentous growth, offering broad insights into the biology, pathogenicity, and evolutionary strategies of a leading fungal pathogen.

## Results

### Divergence in function among *C*. *albicans* Flo8-Mfg1-Mss11 complex members

**Fig 1:**
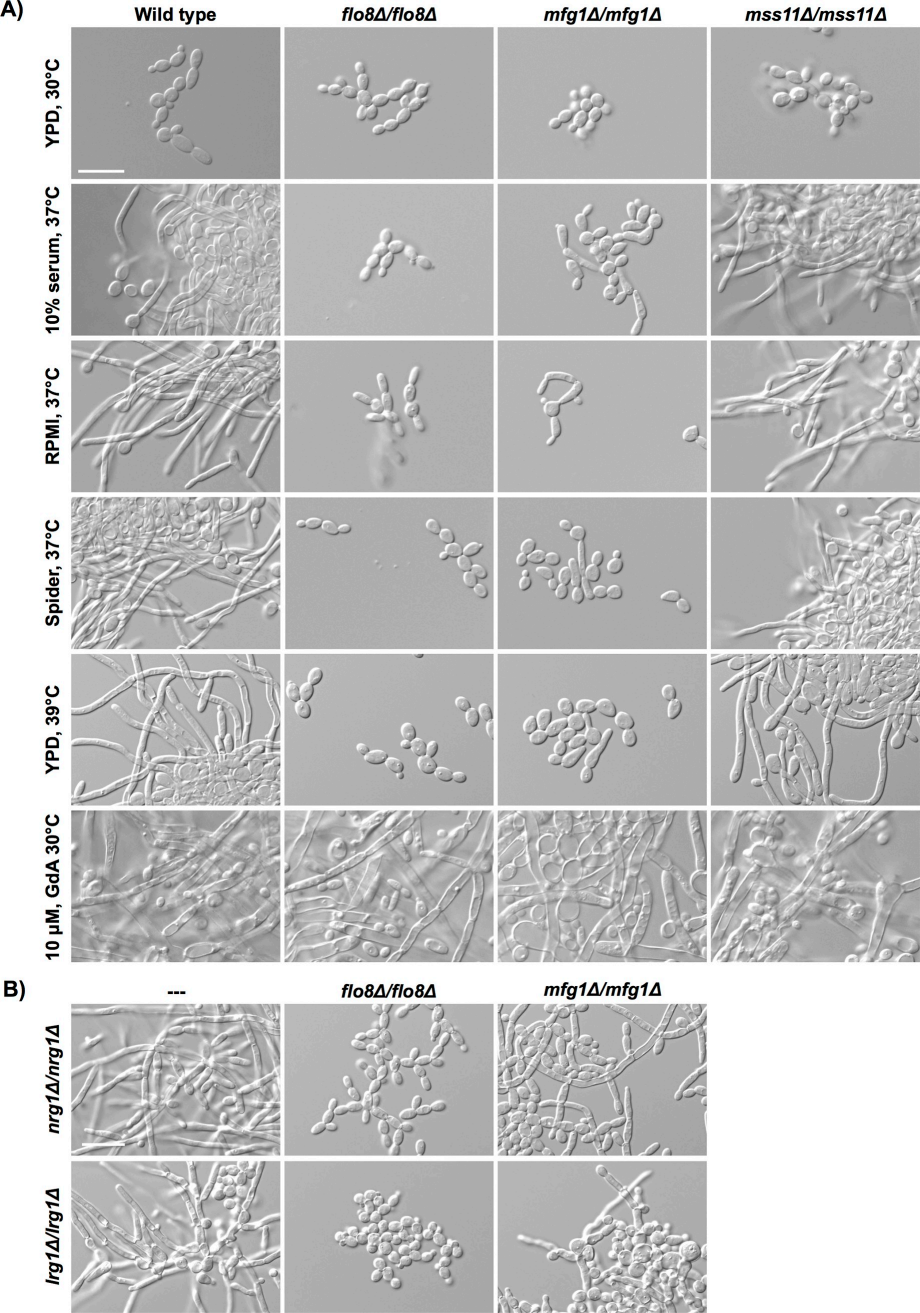
Deletion of genes encoding *C*. *albicans* Flo8-Mfg1-Mss11 complex members has distinct impacts on filamentation induced by diverse cues. **A)** *C*. *albicans flo8Δ/flo8Δ* and *mfg1Δ/mfg1Δ* mutants are largely blocked in filamentation in response to most cues, except in response to Hsp90 inhibition by treatment with geldanamycin (GdA). In contrast, a *C*. *albicans mss11Δ/mss11Δ* mutant was able to filament in response to all cues tested. Cells were grown in the conditions indicated, and were imaged after 3.5 hours, except for those grown in the presence of GdA, which were imaged after 24 hours. Scale bar is 20 μm. **B)** *FLO8* is required for filamentation induced by deletion of the negative regulators of filamentation, *NRG1* or *LRG1*, while *MFG1* is not required. Cells were grown in YPD for 5 hours at 30°C. Scale bar is 20 μm.

Flo8 and Mfg1 are critical regulators of filamentation in *C*. *albicans*, and they physically interact with each other and with the transcriptional regulator Mss11 under basal conditions [26]. However, the mechanisms by which this complex regulates morphogenesis in *C*. *albicans* remain largely enigmatic. To characterize the role of the Flo8-Mfg1-Mss11 complex in the yeast-to-hyphal transition, we examined the morphology of *C*. *albicans flo8Δ/flo8Δ*, *mfg1Δ/mfg1Δ*, and *mss11Δ/mss11Δ* mutants in response to a variety of filament-inducing cues. Similar to previous reports [28], we observed that *flo8Δ/flo8Δ* mutants were completely blocked in filamentous growth in response to 10% serum, RPMI, Spider medium, or high temperature (Fig 1A). However, this strain was capable of forming robust filaments in response to Hsp90 inhibition with geldanamycin (Fig 1A). Similar to the *flo8Δ/flo8Δ* mutant, the *mfg1Δ/mfg1Δ* mutant was largely blocked in filamentation in the majority of cues examined, but was capable of filamenting in response to Hsp90 inhibition (Fig 1A). Notably, restoration of one allele of *MFG1* [26] or *FLO8* (S1 Fig) restored the capacity of the respective nulls to filament. In contrast to previous reports [29], no observable defect in filamentous growth was observed in two independently generated *MSS11* mutants (Fig 1A and S2 Fig). As Flo8 and Mfg1 play central roles in filamentation induced by diverse cues, we also assessed their impact on filamentation induced by deletion of the repressors of filamentation, *NRG1* [30] and *LRG1* [25]. Strikingly, *FLO8* was required for filamentation induced by loss of either *NRG1* or *LRG1*, while *MFG1* was largely dispensable (Fig 1B). Collectively, these results highlight that although Flo8 and Mfg1 are important for filamentation, mutants lacking these regulators are capable of polarized growth under specific environmental conditions and complex members have distinct roles in *C*. *albicans* filamentation.

**Fig 2:**
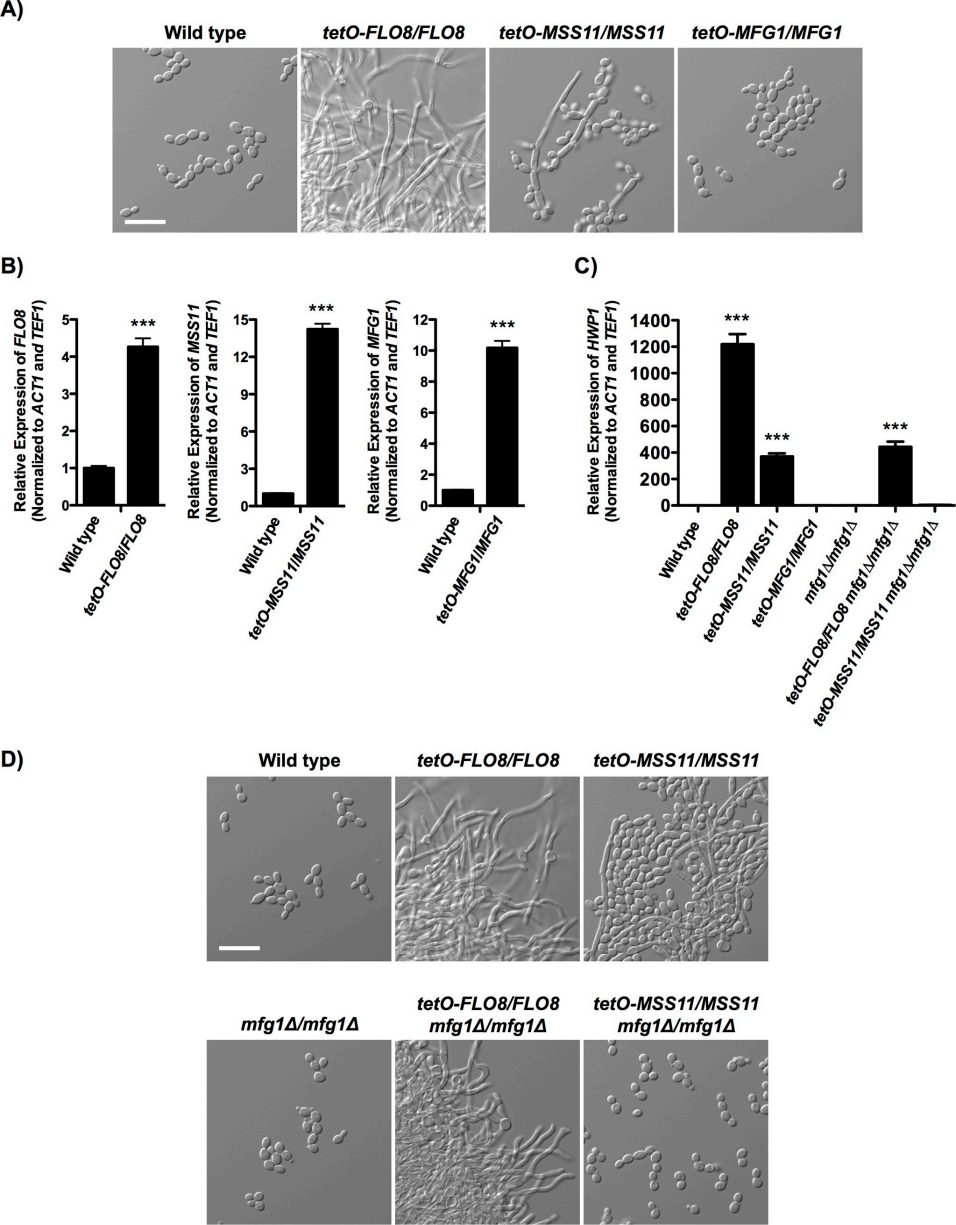
Overexpression of *FLO8* or *MSS11* results in filamentous growth in the absence of an inducing cue, but overexpression of *MFG1* does not. **A)** *FLO8*, *MSS11* or *MFG1* were overexpressed by replacing the native promoter of one allele with a tetracycline-repressible promoter, *tetO*. Cells were grown in YPD at 30°C for 6 hours. Scale bar is 20 μm. **B)** Individual overexpression of genes encoding each complex member was achieved by replacing the native promoter of one allele with a tetracycline-repressible promoter, *tetO*, which in the absence of tetracycline drives constitutive expression of the target gene. Cells were grown in YPD at 30°C for 3 hours. Transcript levels were normalized to *ACT1* and *TEF1* and error bars represent standard error of technical triplicates. Assays were performed in biological duplicate. Asterisks indicate P< 0.0001 (***) relative to the wild type (two tailed unpaired t-test). **C)** Filamentation was quantified by monitoring expression of the filament-specific transcript *HWP1* using qRT-PCR, and normalizing to *ACT1* and *TEF1*. Cells were grown for 4 hours in YPD at 30°C. Error bars represent standard error of technical triplicates as is representative of two biological replicates. Asterisks indicate P < 0.0001 (***) relative to parental strain (one-way ANOVA, Bonferroni Multiple Comparison Test). **D)** Filamentation induced by overexpression of *FLO8* is largely independent of Mfg1, whereas filamentation induced by overexpression of *MSS11* is completely dependent on Mfg1. Cells were grown in YPD at 30°C for 6 hours. Scale bar is 20 μm.

Given that complex members are important to enable *C*. *albicans* filamentation in response to many cues, we next assessed if overexpression of any single component was sufficient to drive the filamentous growth program. Notably, overexpression of *S*. *cerevisiae FLO8* or *MSS11* has previously been shown to result in hyperfilamentous growth [31,32]. We overexpressed each of the three transcriptional regulators individually in *C*. *albicans* by replacing the native promoter of one allele with a tetracycline-repressible promoter, *tetO*, which drives strong and constitutive expression of the target gene in the absence of tetracycline (Fig 2A and 2B). Overexpression of *FLO8* was sufficient to induce robust filamentous growth in the absence of an inducing cue, and resulted in a ~1,200-fold induction of the filament-specific transcript *HWP1* relative to the wild-type strain (Fig 2A and 2C). This is reminiscent of other reports in which overexpression of *C*. *albicans FLO8* resulted in wrinkly colony formation in 5% CO_2_ [33]. Consistent with previous reports [29], overexpression of *C*. *albicans MSS11* was sufficient to induce some filamentation in the absence of an inducing cue and resulted in a 350-fold increase in expression of *HWP1* relative to the wild-type strain (Fig 2A and 2C), highlighting that although this regulator is not necessary for filamentation, it does play a role in regulating this morphogenetic trait. Notably, the filaments induced by overexpression of *FLO8* appeared distinct from those resulting from *MSS11* overexpression, where the former resembled true hyphae, and the latter included many yeast-form cells, suggesting distinct mechanisms. Intriguingly, overexpression of *C*. *albicans MFG1* did not result in filamentous growth in the absence of a filament-inducing cue, nor did it result in an induction of *HWP1* (Fig 2A and 2C). This further supports our model that Flo8, Mfg1, and Mss11 have distinct roles in governing the yeast-to-hyphal transition.

Next, we examined if filamentation induced by overexpression of *FLO8* or *MSS11* was contingent upon Mfg1, given that *MFG1* is required for filamentation in response to several inducing cues (Fig 1A). We replaced the native promoter of one allele of *FLO8* or *MSS11* with the *tetO* promoter, as above, in an *mfg1Δ/mfg1Δ* background (S3 Fig). Overexpression of *FLO8* resulted in robust filamentous growth even in the absence of *MFG1*, and *HWP1* transcript levels were induced by ~450-fold compared to the wild type (Fig 2C and 2D); this is less than half of the increase in *HWP1* that was observed in the presence of *MFG1*, suggesting that Mfg1 promotes filamentation induced by Flo8. In contrast, overexpression of *MSS11* was unable to drive filamentation in the absence of Mfg1, and did not result in induction of *HWP1* (Fig 2C and 2D). Taken together, although Flo8, Mfg1, and Mss11 can exist as a complex in *C*. *albicans* under basal conditions, they also have distinct functions that influence morphogenesis.

### *C*. *albicans* Mfg1 partially complements the activity of its *S*. *cerevisiae* ortholog

**Fig 3:**
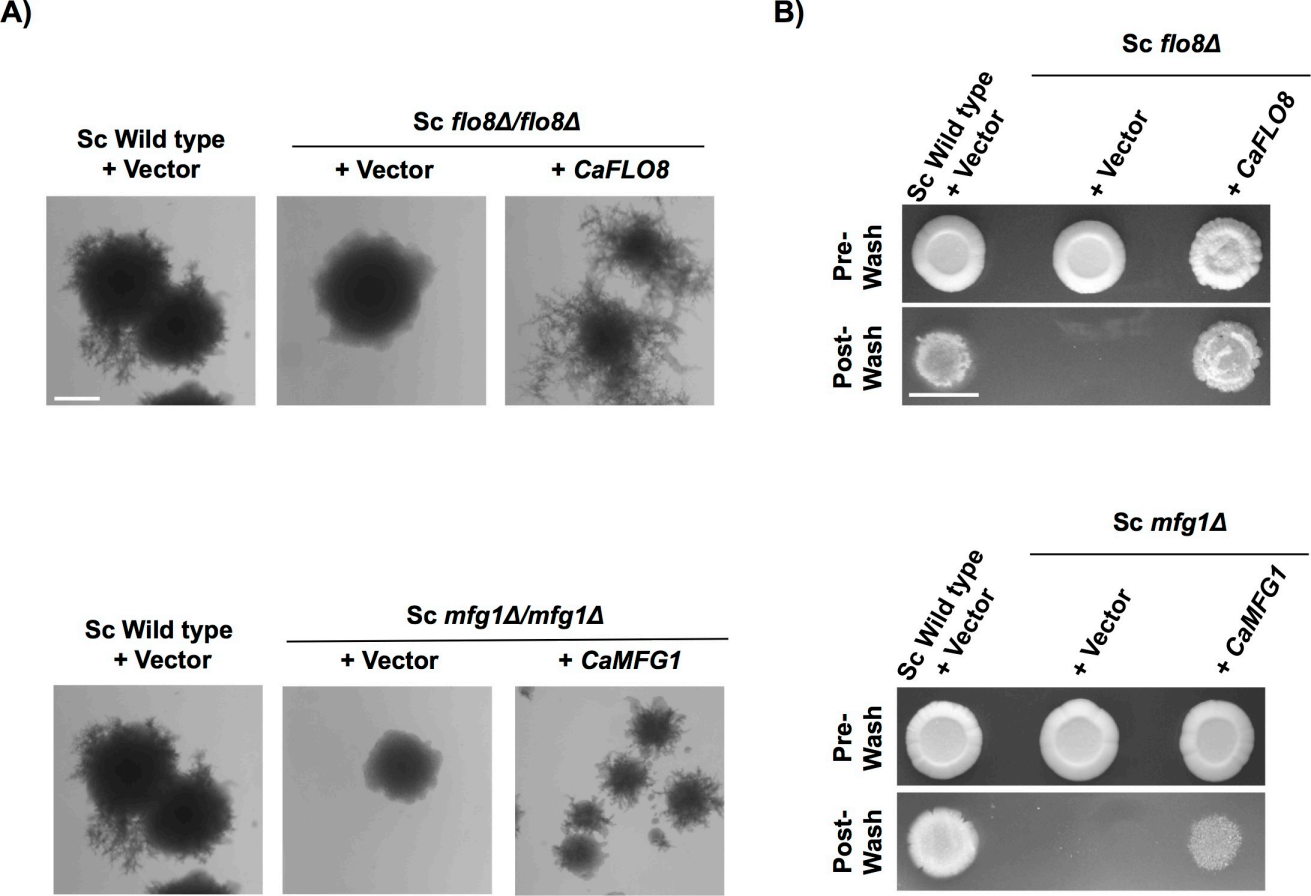
*C*. *albicans* Mfg1 partially complements the activity of its *S*. *cerevisiae* ortholog. **A)** *C*. *albicans FLO8* or *MFG1* were expressed in diploid cells of the filamentation-competent *S*. *cerevisiae* Σ1278b strain (Sc Wild type) and in the *S*. *cerevisiae* strains lacking the relevant orthologs to assess the ability of these complex members to complement the block in *S*. *cerevisiae* diploid pseudohyphal growth. Cells were grown on SLAD agar plates and incubated at 30°C for 11 days. Scale bar is 1.0 mm. **B)** *C*. *albicans FLO8* or *MFG1* were expressed in *S*. *cerevisiae* Σ1278b haploid cells (Sc Wild type) to assess their abilities to complement lack of *FLO8* or *MFG1* in *S*. *cerevisiae* haploid invasive growth. Cells were spotted onto YPD agar plates and incubated at 30°C for 4 days at which point they were washed with water and images of the plate were taken with a Canon Power Shot A610. Scale bar is 1.0 cm.

Given our findings that *C*. *albicans* Flo8 and Mfg1 are key for filamentation in response to most cues (Fig 1A), we assessed if they displayed a conserved function with those complex members in *S*. *cerevisiae*. *C*. *albicans FLO8* has been previously demonstrated to complement the filamentous growth defect of *S*. *cerevisiae* haploid *flo8Δ* and diploid *flo8Δ/flo8Δ* mutants [28]. Here, we cloned *C*. *albicans MFG1* or *FLO8* as a control, into an expression vector and expressed the constructs in the filamentation-competent *S*. *cerevisiae* background Σ1278b. Diploid cells were plated on nitrogen-limiting SLAD medium to monitor pseudohyphal growth. When cells expressed the empty vector, the wild-type diploid formed pseudohyphae and the *flo8Δ/flo8Δ* and *mfg1Δ/mfg1Δ* mutants were blocked in pseudohyphal growth (Fig 3A). As previously described, pseudohyphal growth was restored in the *flo8Δ/flo8Δ* mutant when *C*. *albicans FLO8* was expressed (Fig 3A), demonstrating that it functionally complements the *S*. *cerevisiae* ortholog. We also observed that *C*. *albicans MFG1* was able to functionally complement a *S*. *cerevisiae* strain lacking *MFG1*, resulting in pseudohyphal growth (Fig 3A). Similarly, expression of *C*. *albicans FLO8* in the corresponding *S*. *cerevisiae* haploid deletion mutant was sufficient to restore haploid invasive growth, while expression of *C*. *albicans MFG1* was able to partially restore invasive growth in the *mfg1* haploid mutant (Fig 3B). Thus, *C*. *albicans* Mfg1 is able to at least partially functionally complement its *S*. *cerevisiae* ortholog.

### Mfg1 functions downstream of PKA to drive filamentation

**Fig 4:**
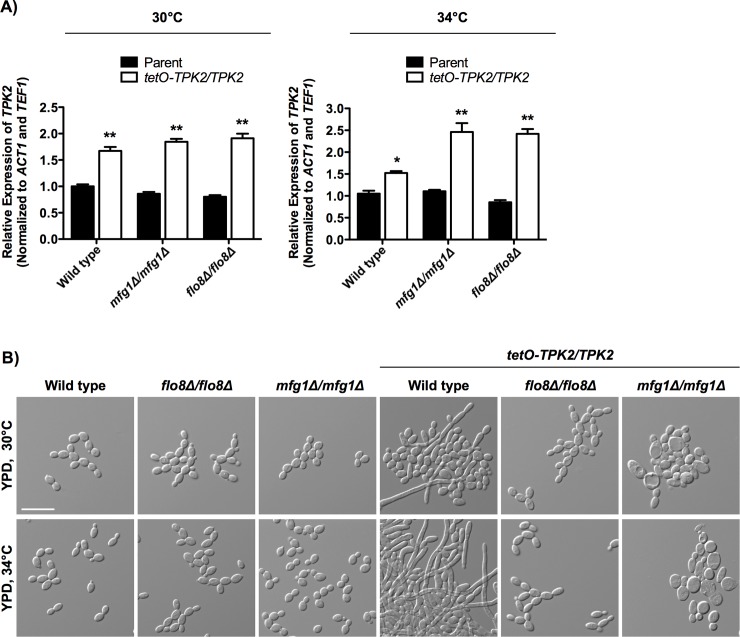
*C*. *albicans* Mfg1 acts downstream of the PKA complex, as does Flo8. **A)** Quantification of *TPK2* expression by qRT-PCR. Overexpression of *TPK2* was achieved by replacing the native promoter of one allele with a tetracycline-repressible promoter, *tetO*. Cells were grown in YPD for either 4 hours (30°C) or 3.5 hours (34°C). *TPK2* transcript levels were monitored using qRT-PCR and normalized to *ACT1* and *TEF1*. Error bars represent standard error of technical triplicates. Assays were performed in biological duplicate. Asterisks indicate P < 0.05 (*) and P < 0.001 (**), relative to parental strain (two-way ANOVA, Bonferroni Multiple Comparison Test). **B)** *TPK2* was overexpressed by replacing the native promoter of one *TPK2* allele with the tetracycline-repressible promoter, *tetO*, in either wild-type, *mfg1Δ/mfg1Δ* or *flo8Δ/flo8Δ* strains. Cells were grown in YPD for 6 hours at either 30°C or 34°C. Scale bar is 20 μm.

In both *S*. *cerevisiae* and *C*. *albicans*, Flo8 functions downstream of PKA to regulate morphogenesis [28,34]. To determine if Mfg1 also acts downstream of the PKA complex in *C*. *albicans*, we overexpressed one of the PKA catalytic subunits, *TPK2*, by replacing the native promoter of one allele of *TPK2* with the tetracycline-repressible promoter, *tetO*, to drive strong constitutive expression in the absence of tetracycline in the wild-type, *flo8Δ/flo8Δ* and *mfg1Δ/mfg1Δ* strains (Fig 4A). Cells were grown in rich medium at 30°C or 34°C, conditions that did not induce filamentous growth in wild-type cells, but caused substantial filamentation when *TPK2* was overexpressed (Fig 4B). As expected, cells remained in the yeast form in the absence of *FLO8* despite overexpression of *TPK2* at 30°C or 34°C (Fig 4A and 4B), confirming that Flo8 acts downstream of PKA. Similarly, overexpression of *TPK2* was insufficient to restore filamentation in the absence of *MFG1* at either temperature (Fig 4A and 4B), suggesting that Mfg1 also acts downstream of PKA to enable *C*. *albicans* morphogenesis. Interestingly, *TPK2* overexpression in the *mfg1Δ/mfg1Δ* background resulted in an enlarged cellular morphology. This demonstrates another phenotypic difference between *flo8Δ/flo8Δ* and *mfg1Δ/mfg1Δ* null mutants.

### Flo8 and Mfg1 possess both shared and distinct transcriptional targets

**Fig 5:**
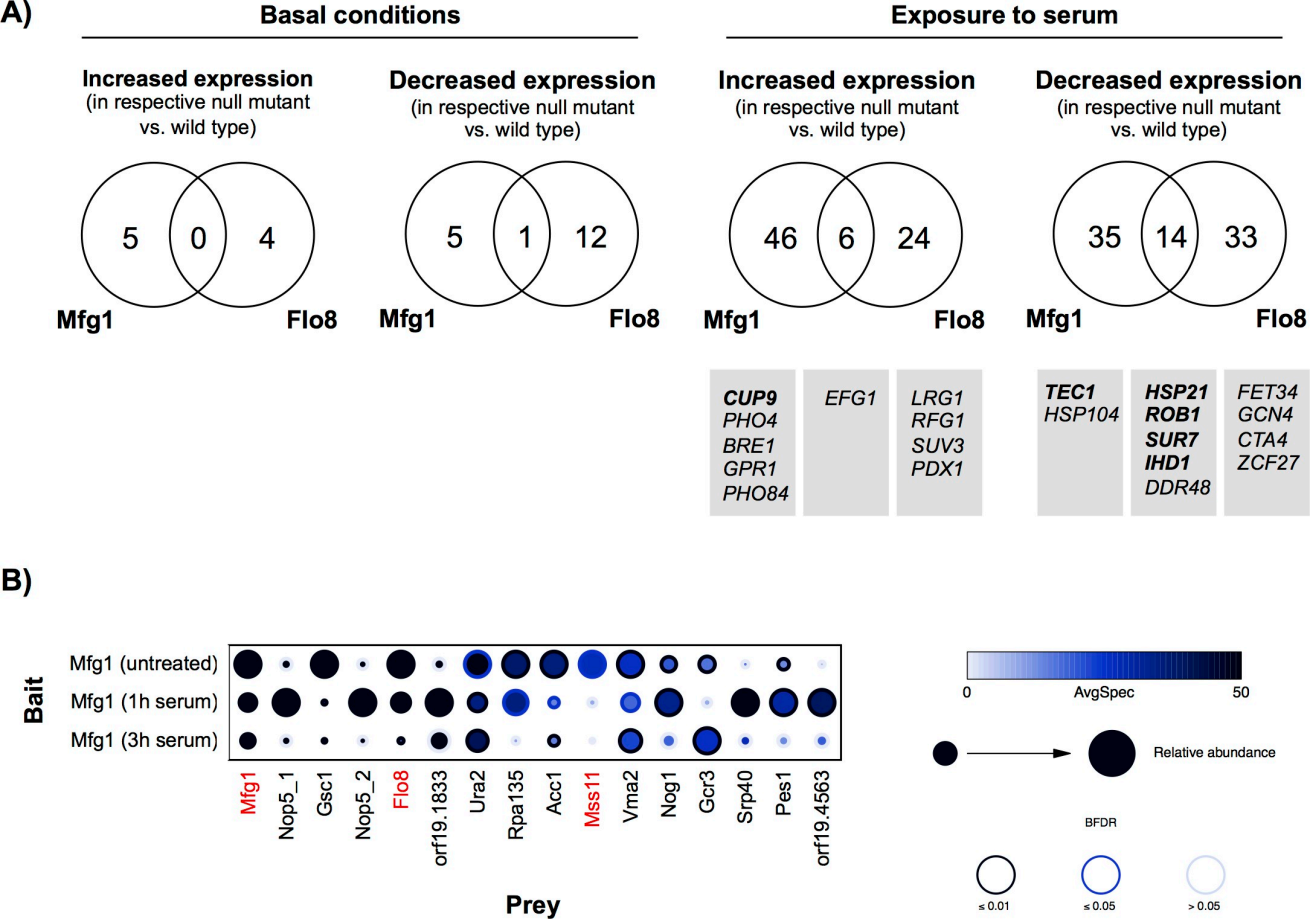
ChIP-chip and transcriptional analyses reveal targets of Mfg1 and Flo8, and AP-MS reveals continued physical interaction under filament-inducing conditions. **A)** Mfg1 and Flo8 bind and regulate overlapping and distinct targets, which are condition specific. Venn diagrams indicate number of genes whose promoters were bound by Mfg1 or Flo8 in filament-inducing conditions (either 1 hour or 3 hours), and for which expression was altered in the respective null mutants at the respective time point. Select targets in serum-treated conditions that are related to filamentation are indicated below the relevant category. **B)** AP-MS was performed with Mfg1-GFP cells grown in untreated conditions, or in the presence of serum at 37°C for 1 hour or 3 hours. Shown are prey proteins with a minimum of 25 detected peptides averaged between two biological replicates, in at least one of the three growth conditions. Mfg1, Flo8, and Mss11 are indicated in red. The separate Nop5 entries correspond to unique BioGrid IDs. Inside circle colour indicates average spectral counts, size of the circle indicates relative protein abundance, and the rim of the circle indicates the Bayesian false discovery rate (BFDR).

Given that perturbation of *C*. *albicans* Flo8 and Mfg1 had distinct consequences on filamentous growth (Fig 1 and Fig 2), we next pursued a global analysis of genes bound and regulated by complex members to further probe their contributions to morphogenesis. Notably, both Flo8 and Mfg1 are localized to the nucleus in basal conditions and upon treatment with serum (S4A Fig). We performed chromatin immunoprecipitation coupled with microarray analysis (ChIP-chip) with strains harboring Flo8 or Mfg1 tagged with a tandem affinity purification (TAP) epitope. Functionality of the TAP-tagged proteins was verified (S4B Fig). DNA binding of Flo8 or Mfg1 was measured in either untreated conditions, or upon exposure to a filament-inducing cue, 10% serum, for one hour or three hours, to identify promoter regions that were bound by these transcriptional regulators (S1 Table). This demonstrated a substantial increase in promoter occupancy of both Flo8 and Mfg1 upon exposure to serum, consistent with their roles as transcriptional regulators of morphogenesis (Fig 5A, S5A Fig and S5B Fig). To identify the Flo8 and Mfg1 targets that are also transcriptionally modulated by these regulators, we performed microarray analysis comparing the gene expression profiles of *flo8Δ/flo8Δ*, *mfg1Δ/mfg1Δ*, and *flo8Δ/flo8Δ mfg1Δ/mfg1Δ* mutants with a wild-type strain under basal and filament-inducing conditions (S2 Table). We noted stark differences in transcriptional regulation by Flo8 and Mfg1 in response to serum, where large sets of genes were specifically altered in expression in either the *flo8Δ /flo8Δ* or *mfg1Δ/mfg1Δ* mutant, or in the double mutant (S5C Fig and S5D Fig). There was a considerable temporal component to the effects on gene expression, where we observed significant differences between the one-hour and three-hour serum-exposed conditions (S5C Fig and S5D Fig).

In order to identify key downstream targets of Flo8 and Mfg1 through which they regulate *C*. *albicans* filamentation, we compared those genes both bound and transcriptionally regulated by Flo8 and Mfg1 (Fig 5A, S3 Table). In basal conditions, we identified only 17 and 11 genes whose promoters were bound by either Flo8 or Mfg1 respectively, and whose expression was altered (Fig 5A). These sets were expanded in the presence of serum, where we identified 77 genes whose promoters were bound by Flo8 and 101 bound by Mfg1 in filament-inducing conditions, and for which expression was altered in the respective null mutants at the respective time point (Fig 5A). Surprisingly, unlike *S*. *cerevisiae* Flo8 and Mfg1 which bind to overlapping targets [26], *C*. *albicans* Flo8 and Mfg1 bind and regulate largely distinct sets of targets (Fig 5A, S5A Fig and S5B Fig, S1 Table), which is striking since Mfg1 does not have a characterized DNA-binding motif. This highlights the unique functions of Flo8 and Mfg1 in regulating gene expression and identifies dynamic transcriptional changes that occur in response to serum.

To further explore the divergence in Flo8 and Mfg1 targets and to identify any potential additional Mfg1-binding partners, we performed affinity purification coupled to mass spectrometry (AP-MS) with C-terminally GFP-tagged Mfg1. Functionality of the tagged protein was verified (S4C Fig). Our previous AP-MS experiments demonstrated that *C*. *albicans* Mfg1 physically interacts with both Flo8 and Mss11 under standard conditions [26]. Here, we expanded the analysis to identify Mfg1 interaction partners in filament-inducing conditions (1 hour or 3 hours of growth in 10% serum at 37°C), and included sonication and benzonase-treatment to aid in the identification of chromatin-bound proteins. In untreated conditions, Mfg1 interacts strongly with Flo8 and Mss11, as expected (Fig 5B, S4 Table). Under filament-inducing conditions, Flo8 continues to interact with Mfg1, suggesting a key role for their interaction. However, interaction with Mss11 is decreased in filament-inducing conditions, consistent with it being dispensable for filamentation (Fig 1A, Fig 5B, S4 Table). Interestingly, while the AP-MS identified other proteins as Mfg1 interactors, there were no additional DNA-binding proteins that could mediate Mfg1 binding to DNA, even in response to serum (Fig 5B, S4 Table).

### Flo8 and Mfg1 cooperatively regulate *TEC1* and *BRG1* expression to promote filamentation, and overexpression of *FLO8* is sufficient to drive an increase in their expression

**Fig 6:**
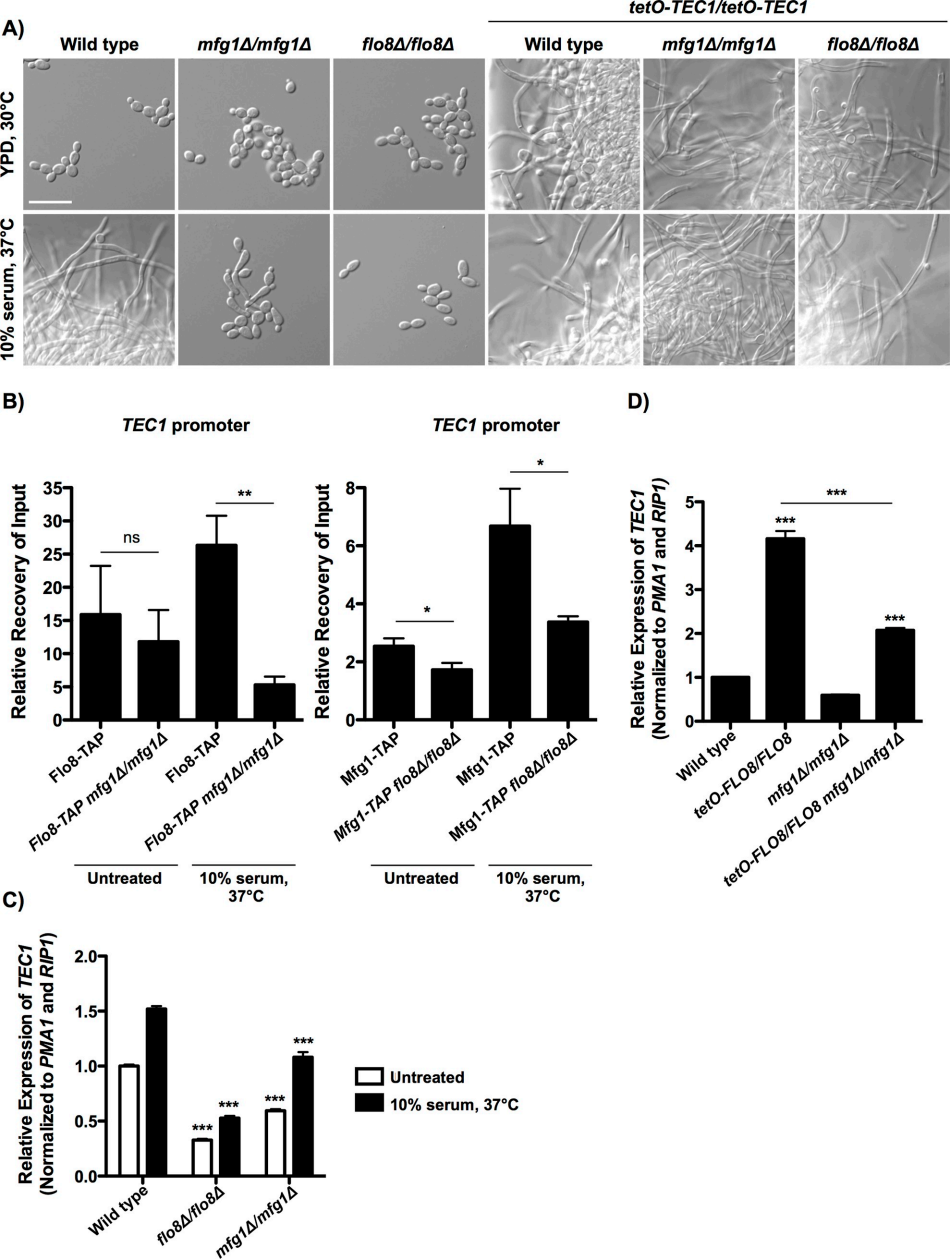
Mfg1 and Flo8 bind to the promoter of *TEC1* to regulate its expression, and overexpression of *FLO8* drives *TEC1* expression, enabling filamentation in the absence of Mfg1. **A)** Overexpression of *TEC1* results in filamentous growth in the absence of *MFG1* or *FLO8*. Cells were grown in YPD at 30°C or in YPD with 10% serum at 37°C for 5 hours. Scale bar is 20 μm. **B)** Binding of Flo8-TAP and Mfg1-TAP to the promoter of *TEC1* was assessed using ChIP-qPCR. Shown is the fold-enrichment over the untagged parental strain, which is set at 1. Asterisks indicate P < 0.01 (**) or P < 0.05 (*), relative to the wild-type parent in the respective condition (two-tailed unpaired t-test). Error bars represent the standard deviation of technical triplicates. Assays were performed in biological duplicate. **C**) *TEC1* expression is reduced in the absence of Flo8 or Mfg1. Cells were grown in YPD at 30°C for 3.5 hours, pelleted and washed, and then transferred to YPD at 30°C or to YPD at 37°C with 10% serum for 1 hour. Transcript levels were monitored using qRT-PCR and normalized to *PMA1* and *RIP1*. Error bars represent standard error of technical triplicates. Assays were performed in biological duplicate. Asterisks indicate P < 0.001 (***), relative to the wild-type strain in each respective condition (two-way ANOVA, Bonferroni Multiple Comparison Test). **D)** *TEC1* expression is increased in strains overexpressing *FLO8*. Cells were grown in YPD at 30°C for 4.5 hours. Transcript levels were monitored using qRT-PCR and normalized to *PMA1* and *RIP1*. Error bars represent standard error of technical triplicates. Assays were performed in biological duplicate. Asterisks indicate P < 0.0001 (***), relative to the respective parental strain, or as indicated (one-way ANOVA, Bonferroni Multiple Comparison Test).

Many of the genes bound and transcriptionally regulated by Flo8 or Mfg1 upon exposure to serum have been implicated in morphogenesis (Fig 5A), suggesting that they could be key downstream targets through which Flo8 and Mfg1 regulate *C*. *albicans* filamentation. We leveraged this data to probe circuitry downstream of the relatively uncharacterized regulator, Mfg1. We initially focused on a subset of genes that were direct targets of Mfg1 and for which expression was altered in the corresponding condition in the *mfg1Δ/mfg1Δ* mutant, including the positive regulators of filamentation *TEC1*, *ROB1*, *HSP21*, *IHD1* and *SUR7*, as well as the negative regulator *CUP9*. We determined if the effectors bound and regulated by Mfg1 were sufficient to promote filamentous growth in the absence of *MFG1* by overexpressing each positive regulator in an *mfg1Δ/mfg1Δ* background using the strong *tetO* promoter, or by deleting negative regulators of filamentation (Fig 6A and S6 Fig). Overexpression of *ROB1*, *HSP21*, *IHD1*, or *SUR7*, or deletion of *CUP9*, did not restore the ability of the *mfg1Δ/mfg1Δ* mutant to filament (S6A Fig and S6B Fig), suggesting that none of these effectors alone are sufficient to modulate Mfg1-mediated filamentation. As previously reported, overexpression of the transcription factor encoded by *TEC1* in an otherwise wild-type background resulted in robust filamentation, even in the absence of an inducing cue [35] (Fig 6A and S6C Fig). Strikingly, overexpression of *TEC1* was sufficient to induce robust filamentation in the absence of Mfg1 (Fig 6A and S6C Fig), suggesting that *TEC1* may be a key regulator of filamentation downstream of Mfg1. Interestingly, *TEC1* overexpression also resulted in robust filamentation in the absence of Flo8 (Fig 6A and S6C Fig), suggesting that it acts downstream of both regulators.

To determine if Flo8 and Mfg1 cooperatively regulate *TEC1* expression, we then examined binding of Flo8 and Mfg1 to the promoter of *TEC1* in the absence of the other regulator. By ChIP-qPCR, we observed increased binding compared to the untagged control of both Flo8 and Mfg1 to the *TEC1* promoter, especially in the presence of serum at 37°C for 1 hour, which was significantly reduced in the absence of the other regulator (Fig 6B). This suggests a functional relationship between Flo8 and Mfg1 at the *TEC1* promoter. To further explore this relationship, we measured transcript levels of *FLO8* and *MFG1*, and protein levels of Flo8-TAP and Mfg1-TAP, in the absence of the other regulator. While deletion of Flo8 or Mfg1 had no effect on transcript level of the other regulator (S7A Fig), deletion of *FLO8* resulted in an approximately 4-fold decrease in Mfg1-TAP levels (S7B Fig). This decrease in Mfg1-TAP protein levels may contribute to the decreased binding observed at the *TEC1* promoter in the absence of *flo8Δ/flo8Δ* (Fig 6B). Nevertheless, Flo8-TAP binding was reduced in the absence of Mfg1 despite the protein levels remaining stable (Fig 6B and S7B Fig), demonstrating a dependency of Flo8 on Mfg1 for binding to target promoters. We further confirmed this observation with another key regulator of filamentation, *BRG1*, where we observed that deletion of either *MFG1* or *FLO8* significantly reduced binding of the other regulator at the *BRG1* promoter (S8A Fig). In addition, we observed that *TEC1* and *BRG1* expression were decreased in the absence of either Flo8 or Mfg1 (Fig 6C and S8B Fig), illustrating that these regulators both bind to the promoters and regulate expression of a subset of key regulators of filamentation.

Considering the key role for Tec1 in regulating filamentation, we further explored its relationship to Flo8 and Mfg1 by examining *TEC1* expression in the *tetO-FLO8/FLO8* overexpression strain. Interestingly, overexpression of *FLO8* drives an approximately 4-fold increase in *TEC1* expression relative to the wild type, which is decreased 2-fold in the absence of Mfg1 (Fig 6D). This is consistent with the fact that overexpression of *FLO8* drives filamentation in the absence of an inducing cue, and to a lesser extent in the absence of *MFG1* (Fig 2C and 2D). We further confirmed this observation by examining *BRG1* expression in the *tetO-FLO8/FLO8* strain, and similarly observed an increase in *BRG1* expression upon overexpression of *FLO8*, which is reduced in the absence of Mfg1 (S8C Fig). Together, this suggests that Flo8 and Mfg1 cooperatively bind to the promoters of key regulators of filamentation, and that overexpression of *FLO8* is sufficient to drive expression of these regulators and induce morphogenesis even in the absence of Mfg1.

### Aneuploidy of chromosome 6 restores filamentous growth in an *mfg1Δ/mfg1Δ* mutant

**Fig 7:**
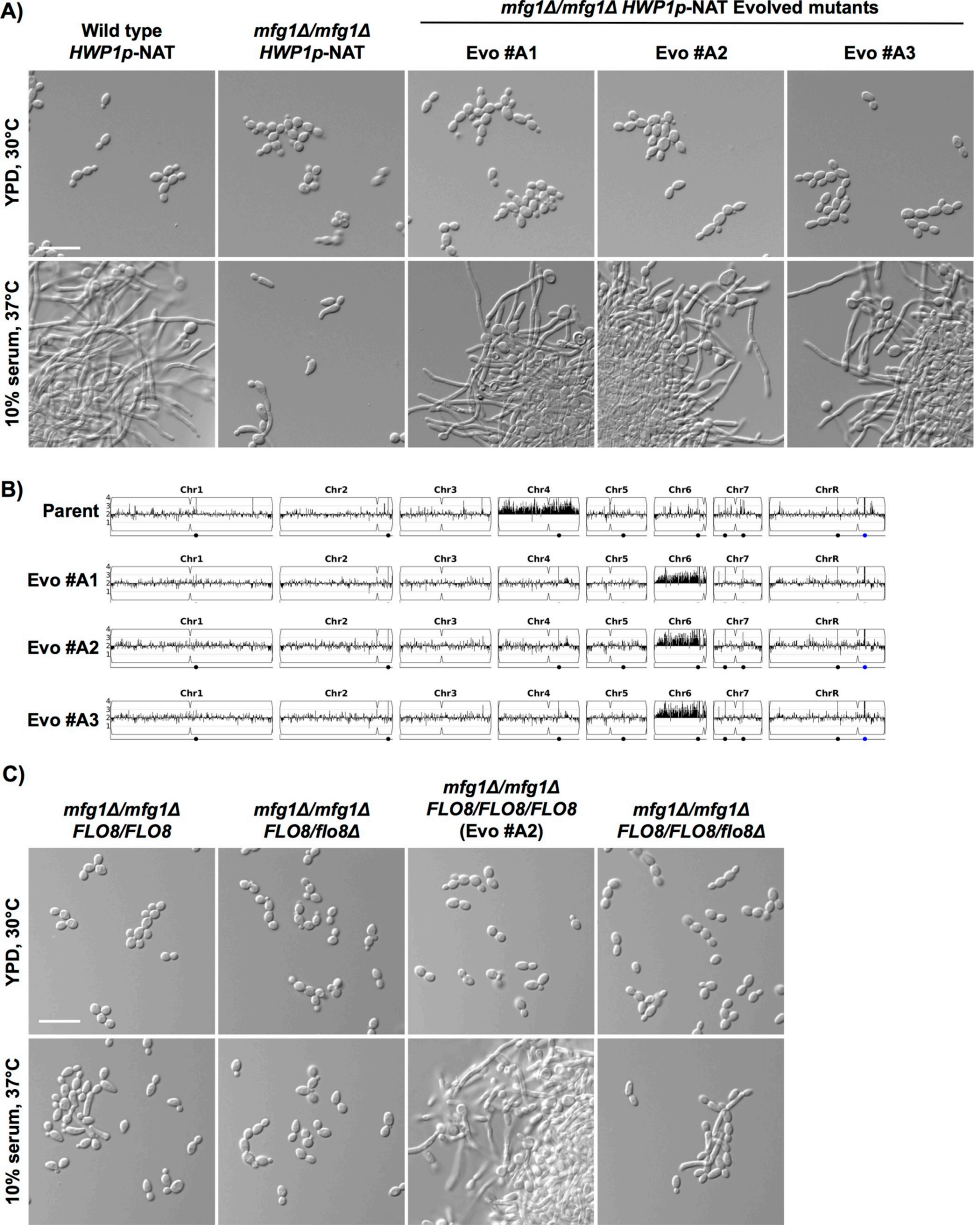
Amplification of chromosome 6 restores the ability of an *mfg1Δ/mfg1Δ* mutant to filament. **A)** Independent colonies from an *mfg1Δ/mfg1Δ* mutant expressing the NAT (nourseothricin) resistance marker from the *HWP1* promoter were plated on solid YPD containing 10% serum and 250 μg/mL NAT and grown at 37°C for 2 days. Shown are three independently evolved mutants that were able to filament in liquid conditions in response to serum (Evo #A1, Evo #A2, and Evo #A3). Cells were grown in YPD at 30°C or in YPD with 10% serum at 37°C for 6 hours. Scale bar is 20 μm. **B)** The genomes of three independently evolved filamentous mutants (Evo #A1, Evo #A2 and Evo #A3) in the *mfg1Δ/mfg1Δ* background were sequenced and profiled for copy number variants using Y_MAP_. **C)** Deletion of one allele of *FLO8* from the evolved mutant Evo #A2 impairs its ability to filament. One allele of *FLO8* was replaced with *CdARG4*, and cells were grown as in A). Scale bar is 20 μm.

To identify additional circuitry downstream of Mfg1 important for *C*. *albicans* filamentation, we turned to an alternative, unbiased approach and employed a novel selection strategy to evolve mutants capable of filamenting in a strain lacking *MFG1*. Our approach employed a dominant nourseothricin (NAT) resistance marker under the control of a filament-specific promoter (*HWP1p*), such that the expression of NAT occurs only when cells are undergoing filamentous growth, thereby enabling selection for filamentation on plates containing NAT. We introduced this system into an *mfg1Δ/mfg1Δ* mutant, and plated cells on filament-inducing conditions of rich medium containing 10% serum with a high concentration of NAT, with incubation at 37°C for two days. We evolved three independent lineages in the *mfg1Δ/mfg1Δ* background with a restored capacity to undergo robust filamentation in liquid medium containing serum as observed after six hours of growth (Fig 7A). Notably, this filamentation phenotype was largely lost after growth in serum for 24 hours, whereas wild-type cells were still filamentous, suggesting that filamentation could not be maintained in the absence of *MFG1* (S9A Fig). This demonstrates that our *HWP1p-NAT* selection strategy provides adequate selective pressure to evolve the capacity for morphogenesis in filamentation-defective mutants.

To determine the genetic basis for this restoration of filamentation in the absence of *MFG1*, we performed whole genome sequencing of the three independently evolved mutants. Although no single nucleotide variants or small insertions or deletions in coding regions were identified that were common to all of the evolved lineages, we did observe a common decrease in copy number of chromosome 4 and an increase in copy number of chromosome 6 relative to the parent (Fig 7B). Upon closer examination, the apparent loss of chromosome 4 was due to a chromosome 4 trisomy in the parental strain, resulting in all evolved lineages having two copies of this chromosome. We therefore focused our analysis on the increased copy number of chromosome 6. While many genes involved in filamentation are located on chromosome 6, a likely candidate responsible for restoring filamentous growth was *FLO8*, as we previously observed that overexpression of *FLO8* was sufficient to promote morphogenesis in the absence of *MFG1* (Fig 2D). Increased expression of *FLO8* in the evolved strains was verified by qRT-PCR (S9B Fig). To determine if the increase in *FLO8* copy number was responsible for restoring filamentation of the *mfg1Δ/mfg1Δ* mutant, we deleted one allele of *FLO8* from one of the evolved filamentous mutants and observed a corresponding decrease in ability of this strain to filament in response to serum (Fig 7C). This suggests that the chromosome 6 amplification may have enabled filamentation in response to serum by increasing the copy number of *FLO8*. Furthermore, we examined *TEC1* and *BRG1* expression in this evolved strain, as we had observed that *FLO8* overexpression results in increased expression of these important regulators of filamentation, even in the absence of Mfg1 (Fig 6D and S8C Fig). Indeed, when grown in filament-inducing conditions, we observe a small but significant increase in both *TEC1* and *BRG1* transcript levels in an *mfg1Δ/mfg1Δ* evolved strain with a chromosome 6 amplification (S9C Fig). Notably, we also attempted to evolve filament-capable mutants in the *flo8Δ/flo8Δ* mutant and the *mfg1Δ/mfg1Δ flo8Δ/flo8Δ mss11Δ/mss11Δ* triple deletion mutant, yet were unable to obtain NAT-resistant colonies when plating cells on medium containing serum and a high concentration of NAT (S9D Fig), confirming that Flo8 is critical for morphogenesis in this experimental context. Together this suggests that Flo8 plays a pivotal role in enabling filamentation even in the absence of *MFG1*, and may do so by driving expression of key regulators of filamentation, including *TEC1* and *BRG1*.

**Fig 8:**
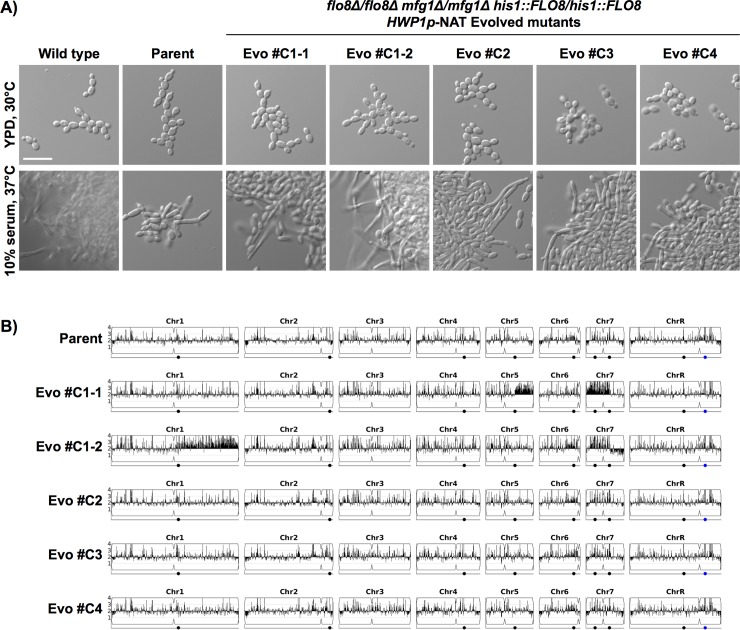
Evolution of filament-capable *mfg1Δ/mfg1Δ* mutants with *FLO8* at a non-native locus is not accompanied by chromosome 6 triplication. **A)** An *mfg1****Δ****/mfg1****Δ*** mutant with *FLO8* located on chromosome 5 (at the *HIS1* locus) and expressing the NAT (nourseothricin) resistance marker under the control of the *HWP1* promoter evolved the ability to filament upon selection on NAT and serum. Mutants Evo #C1-1 and Evo #C1-2 arose from the same overnight culture but showed distinct phenotypes in their ability to filament at later time points. Mutants Evo #C1, C2, C3, and C4 are all independently generated. Cells were grown in YPD at 30°C or in YPD with 10% serum at 37°C for 6 hours. Scale bar is 20 μm. **B)** The genomes of five evolved filamentous mutants in the *mfg1****Δ****/mfg1****Δ*** background were sequenced and profiled for copy number variants using Y_MAP_.

To further explore if *FLO8* amplification underpins this adaptive mechanism to restore filamentation by chromosome 6 amplification, we engineered a strain in which both alleles of *FLO8* were deleted from the endogenous location on chromosome 6 and integrated at a distinct location in the genome, on the right arm of chromosome 5. To confirm that *FLO8* in a new chromosomal context is functional, we verified expression of two genes whose expression increase in the *flo8Δ/flo8Δ* null and *mfg1Δ/mfg1Δ flo8Δ/flo8Δ* double mutant (S2 Table), but are restored to wild-type levels when *FLO8* is integrated on chromosome 5 (S10A Fig). Using our established selection regime, we successfully evolved five lineages in the *mfg1Δ/mfg1Δ* background with *FLO8* located on chromosome 5 that were capable of undergoing filamentation upon growth in liquid medium containing serum (Fig 8A), with many but not all reverting to yeast form growth after 24 hours (S10B Fig). Whole genome sequencing revealed that none of the newly evolved lineages possessed an aneuploidy of chromosome 6 (Fig 8B), providing evidence that the presence of *FLO8* on chromosome 6 was an important determinant of aneuploidy formation in the original lineages (Fig 7). Consistent with the importance of *FLO8* in aneuploidy formation, one of the newly evolved lineages acquired an amplification of the right arm of chromosome 5 (Evo #C1-1), on which *FLO8* was located in this background (Fig 8B). A significant increase in *FLO8* expression in this evolved lineage was verified by qRT-PCR (S10C Fig). Intriguingly, the other newly evolved lineages acquired distinct chromosomal alterations that were independent of *FLO8* copy number, including amplification of the right portion of chromosome 1 (Evo #C1-2), amplification of the left portion of chromosome 7 (Evo #C1-1 and Evo #C1-2), and loss of the right portion of chromosome 7 (Evo #C1-2) (Fig 8B). Interestingly, *NRG1* is located on the right arm of chromosome 7, and deletion of *NRG1* allows filamentous growth in an *mfg1Δ/mfg1Δ* mutant (Fig 1B), suggesting that reduced *NRG1* dosage might be an adaptive mechanism to promote filamentation in this evolved strain. In addition to chromosomal alterations, a few SNPs were identified in the evolved lineages, including in the predicted kinase gene *YAK1* and upstream of the putative transcription factor gene *HOT1* (S5 Table). Together, these results suggest that amplification of *FLO8* is a major driving force of aneuploidy formation in order to restore filamentation in the absence of *MFG1*, and emphasizes that additional factors remain to be discovered.

## Discussion

Fungal species rely on morphogenetic transitions for diverse biological processes, including reproduction, stress adaptation and virulence [2,3]. The transcriptional regulators Flo8, Mfg1 and Mss11 work as a complex in the model yeast *S*. *cerevisiae* to control the expression of genes that enable filamentous growth [26]. Although these regulators are conserved in *C*. *albicans*, as is their physical interaction under basal conditions, we established here that Flo8, Mfg1 and Mss11 possess divergent roles in regulating *C*. *albicans* filamentation (Fig 1 and Fig 2). Genome scale analyses supported the model that although Flo8 and Mfg1 are capable of acting in concert to regulate gene expression, the majority of their transcriptional targets are distinct and environmentally contingent (Fig 5 and S5 Fig). Moreover, we described a novel approach for mapping genetic circuitry important for *C*. *albicans* filamentation, and discovered that increasing *FLO8* copy number can drive aneuploidy formation to restore morphogenesis in the absence of *MFG1* (Fig 7). Thus, we reveal complex genetic circuitry through which Flo8, Mfg1 and Mss11 regulate filamentation, and implicate a new facet of genomic plasticity in pathogen adaptation.

A global analysis exploring regulators of filamentation in *S*. *cerevisiae* and *C*. *albicans* identified the previously uncharacterized Mfg1 as a core regulator of morphogenesis in both species, and showed that it physically interacts with transcriptional regulators Flo8 and Mss11 [26]. In *S*. *cerevisiae*, these three proteins act in concert to enable pseudohyphal growth, biofilm formation and invasive growth through transcriptional regulation of target genes [26]. Genome-scale analyses demonstrated that deletion of any component of this complex largely abrogated binding of other complex members to target genes [26], highlighting the functional interdependence of these transcriptional regulators. In contrast, we identified considerable divergence in complex member function in *C*. *albicans*. Although both Flo8 and Mfg1 were required for filamentous growth, Mss11 was dispensable for hyphal formation in response to any cue that we tested, and its physical interaction with Mfg1 was reduced in filament-inducing conditions (Fig 1 and Fig 5B). Although Flo8 and Mfg1 target largely different genes, they do cooperatively bind to a subset of key regulators of filamentation, including *TEC1* and *BRG1*. Mfg1 and Flo8 binding to these promoters is reduced in the absence of the other regulator (Fig 6B, S8A and S10D Figs), leading to reduced expression of targets such as *TEC1* and *BRG1* (Fig 6C and S8B Fig). However, overexpression of *FLO8* was sufficient to result in increased expression of *TEC1* and *BRG1*, resulting in filamentous growth even in the absence of Mfg1 (Fig 2, Fig 6D, S8C Fig, and S10D Fig), and suggesting that Mfg1 is dispensable for Flo8 binding to at least a subset of key targets or that overexpression of *FLO8* can compensate for the loss of Mfg1. Although Flo8 was required for filamentation in response to loss of the filamentation repressors Nrg1 and Lrg1, Mfg1 was dispensable, highlighting another distinct facet of their consequences on morphogenesis. Interestingly, *mfg1Δ/mfg1Δ* mutants resembled mutants lacking Eed1, a regulator of hyphal extension, which form germ tubes after a short exposure to filament-inducing cues, but revert to yeast after prolonged exposure to the inducing cue [36]. This suggests that Mfg1 may be a key regulator of hyphal extension as opposed to initiation. Together, these findings support evolutionary rewiring of complex member function compared with that observed in *S*. *cerevisiae*.

Experimental evolution has been used extensively to investigate circuitry governing microbial drug resistance, virulence and filamentation. Such studies have revealed that aneuploidies are a prevalent adaptive mechanism in fungi, involving ploidy gain or reduction, upon exposure to stressful environmental conditions [37]. The acquisition of aneuploidies in *C*. *albicans* upon exposure to azoles, the most widely deployed class of antifungal, occurs readily in both *in vitro* experimental evolution experiments, as well as in clinical settings [38–40]. Most common is the formation of an isochromosome on the left arm of chromosome 5 (i(5L)), which increases the copy number of the azole drug target gene *ERG11*, as well as a gene encoding a transcriptional regulator of an efflux pump, *TAC1* [41]. Experimental evolution has also identified adaptations that enable filamentation of non-filamentous *Candida* within mammalian macrophages, where filamentation is favoured as it enables host cell escape [42,43]. A single nucleotide polymorphism in the chitin synthase gene *CHS2* was found to restore filamentation in *Candida glabrata* [43], and a mutation in *SSN3*, encoding a component of the Mediator complex, facilitated filamentation in a non-filamentous *C*. *albicans* strain [42]. We employed a novel selection method to restore morphogenesis in non-filamentous *C*. *albicans* mutants by placing a dominant NAT resistance cassette under the control of the filament-specific *HWP1* promoter. A key advantage of this strategy is that it can be easily applied to diverse genetic backgrounds and environmental conditions. With this approach, we restored filamentous growth in an *mfg1****Δ****/mfg1****Δ*** mutant and identified an increase in chromosome 6 copy number as a mechanism to enable filamentation (Fig 7). Notably, recent findings identified a chromosome 6 trisomy in a parasexual isolate with increased virulence and capacity to filament [44]. Furthermore, preferential amplification of *C*. *albicans* chromosome 6 has been identified upon passage in a murine model of oropharyngeal candidiasis [45], suggesting that amplification of chromosome 6 is also important for adaptation *in vivo*. Analogous adaptive strategies have been reported in *S*. *cerevisiae*, where the gain of a single chromosome was sufficient to transition between smooth and fluffy colony morphologies [46]. Although genomic plasticity has been associated with altered morphologies in fungal pathogens [45,47–52], our work provides the first example of aneuploidy formation as a causative mechanism enabling filamentous growth in the context of *C*. *albicans* experimental evolution.

As the frequency and severity of fungal infections continue to climb, the necessity for novel therapeutic interventions is of utmost importance. Given the limited number of antifungal classes uncovered by traditional approaches of targeting essential proteins, targeting regulators of pathogen-specific virulence traits as a therapeutic strategy is attracting increasing interest [15]. For *C*. *albicans*, the transition between yeast and hyphae is of particular relevance, as both forms are required for virulence. Many small molecules with antifungal properties also have profound effects on fungal dimorphism, including the azole class of antifungals [53], compounds that target the molecular chaperone Hsp90 [54], the promiscuous protein kinase inhibitor staurosporine [55], the natural product beauvericin [56], and the broad-spectrum metal chelator DTPA [57]. Various other small molecules have also been described as able to modulate the transition between yeast and hyphae [58]. Although large-scale chemical biology screens typically monitor pathogen viability to reveal bioactive molecules, recently a screen to identify inhibitors of *C*. *albicans* adhesion uncovered filastatin, an inhibitor of hyphal formation [59]. Analogous approaches using expanded libraries of chemical matter have the potential to reveal additional small molecules capable of attenuating fungal virulence. Intriguingly, targeting specific aneuploidies with small molecules has also been described as a potential strategy, specifically to treat azole-resistant *C*. *albicans* harbouring (i(5L)) [60]. Thus, exploring circuitry governing *C*. *albicans* filamentation has the potential to reveal new strategies to cripple fungal pathogens and also to uncover fascinating biological insights into adaptive mechanisms governing key virulence traits.

## Materials and methods

### Strains and culture conditions

Archives of *C*. *albicans* and *S*. *cerevisiae* strains were maintained at −80°C in rich medium (YPD) or in synthetic defined (SD) medium with 25% glycerol. YPD was prepared as follows: 1% yeast extract, 2% bactopeptone, 2% glucose, with 2% agar for solid medium. SD was prepared as follows: 6.7 g/L yeast nitrogen base, 2% glucose, supplemented with amino acids as necessary. Strains were propagated in YPD or SD medium as required. Spider medium was prepared as previously described [61]. RPMI was prepared as follows: 10.4 g/L RPMI-1640, 3.5% MOPS, 2% glucose, supplemented with an additional 5 mg/mL histidine as required, pH 7. Synthetic low ammonia dextrose (SLAD) solid medium plates supplemented with 15 μM leucine and 10 μM histidine were prepared as previously described [26,62]. Geldanamycin was obtained from LC laboratories, G-4500, and was dissolved in DMSO. Heat-inactivated newborn calf serum (Gibco) was used in YPD at 10%. All strains used in this study are listed in S5 Table and construction is described in S1 Text. To select for nourseothricin (NAT)-resistant mutants, NAT (Jena Bioscience) stock solution was prepared in water at a concentration of 250 mg/mL and YPD plates were supplemented with 150 μg/mL NAT. The *SAP2* promoter was induced to drive expression of the FLP recombinase to excise the NAT marker cassette [63,64]. All plasmids used in this study are listed in S6 Table and all oligonucleotide sequences used in this study are included in S7 Table.

### Microarray profiling

Each microarray experiment was conducted in triplicate. Cells were grown overnight in YPD at 30°C, diluted to OD_600_ of 0.2, and grown to mid-log phase, at which point 10% serum was added and cultures were moved to 37°C for 1 or 3 hours. Cultures were pelleted at 3000 rpm for 5 min and frozen overnight at –80°C. RNA was isolated using the QIAGEN RNeasy kit and RNasefree DNase (QIAGEN). Microarray experiments were performed essentially as described, using a high-density tiling array containing 240,798 unique 60-mer probes [65,66]. Briefly, 20 μg of RNA was reverse transcribed using Superscript III Reverse Transcriptase (Invitrogen) and oligo(dT)21 in the presence of Cy3- or Cy5-dCTP (Invitrogen). Template RNA was degraded using 2.5 units RNase H (USB) and 1 μg RNase A (Pharmacia), incubated at 37°C for 15 min. Labeled cDNA was purified with a QIAquick PCR Purification Kit (QIAGEN). The hybridization was carried out with DIG Easy Hyb Solution (Roche Diagnostics) containing 0.45% salmon sperm DNA and 0.45% yeast tRNA at 42°C for 24 hours in a SlideBooster Hyb chamber SB 800 (Advalytix, Brunnthal, Germany) with regular microagitation. The slides were washed once in 1.0% SSC (0.15 M NaCl and 0.015 M sodium citrate) with 0.2% SDS at 42°C for 5 min; twice in 0.1% SSC with 0.2% SDS at 42°C for 5 min; and once in 0.1% SSC at 24°C for 5 min, followed by four rinses in 0.1% SSC. The microarray slides were air dried before being scanned using a ScanArray Lite microarray scanner (Perkin Elmer). Microarray data were analyzed with GeneSpring GX v7.3 (Agilent Technologies), and genes with statistically significant (*P*<0.05) changes in transcript abundance of ≥1.5-fold were identified with a volcano plot and compared to other lists of significantly modulated genes. Data are accessioned at NCBI GEO GSE117477.

### Whole-genome location profiling by ChIP-chip

ChIP-chip was performed as previously described [65,66]. In brief, binding locations were determined in duplicate ChIP-chip experiments using a high density tiling array containing 240,798 unique 60-mer probes. Fluorescence intensities were quantified using ImaGene software 9.0 (BioDiscovery Inc.), background corrected, and normalized for signal intensity (Lowess normalization). The significance cut-off was determined using the distribution of log-ratios for each factor. It was set at two standard deviations from the mean of log transformed fold enrichments (cut off log ratio of 0.4). ORFs with a binding peak within the 1500 bp 5’ region were identified as targets. Values shown are an average of two biological replicates of tagged and mock constructs. Data are accessioned at NCBI GEO GSE117477.

### Whole genome sequencing

To select for filamentous mutants, 4x10^7^ cells from independent overnights were plated on YPD+10% serum +250 μg/mL NAT. NAT resistant colonies appeared after 2 days at 37°C. Genomic DNA was isolated with phenol chloroform, as described previously [67]. Libraries were prepared using the NexteraXT DNA Sample Preparation Kit following the manufacturer’s instructions (Illumina). Libraries were purified with AMPure XP beads (Agencourt) and library concentration was quantified using a Bioanalyzer High Sensitivity DNA Chip (Agilent Technologies) and a Qubit High Sensitivity dsDNA fluorometric quantification kit (Life Technologies). DNA Libraries were sequenced using paired end 2x250 flow cells on an Illumina MiSeq (Creighton University). Copy number variation was visualized using Y_MAP_ [68]. The sequence data is publicly available on the NCBI Sequence Read Archive with accession number SRP124459.

### ChIP-qPCR

Flo8-TAP and Mfg1-TAP binding at the *BRG1* and *TEC1* promoters were analyzed by ChIP-qPCR, as described in reference [69], with the following modifications. Cells were grown in YPD to middle-late log phase (OD_600_ 4–5), collected by centrifugation and reinoculated to OD_600_ of 1 in 50 mL pre-warmed 30°C YPD or 75 mL 37°C YPD with 10% new-born calf serum (Gibco) and grown for 1 hour before fixation with 1% formaldehyde for 20 minutes. Cells were lysed by bead beating for 5 x 1 minute, and chromatin was sheared by probe sonication (40% amplitude) for 4 x 16 seconds and in a Bio-disruptor water bath sonicator (high setting; 30 seconds on/ 30 seconds off) for 4 x 5 minute intervals. 300 μL of sonicated cell lysates were incubated with 20 μL rabbit IgG agarose beads (Sigma) at 4°C overnight. Beads were extensively washed with wash buffer, deoxycholate buffer and TE. Final ChIP products were eluted and reverse-crosslinked in TE-SDS buffer and purified by PCR purification column (Qiagen). An untagged parental strain (SN95) was tested in parallel as a control, where the tagged cells grown in untreated or hyphal-inducing conditions were normalized to the untagged parents grown in either untreated or hyphal-inducing conditions, respectively. Data analyses were performed similarly as described in reference [69]. Binding was assessed by qPCR using primers oLC7051/oLC7054 (*BRG1*) and oLC7371/oLC7372 (*TEC1*).

### Immunoblotting

Immunoblotting to monitor Flo8-TAP and Mfg1-TAP protein levels was performed as described in reference [69]. Crude cell lysates were resolved on 6% SDS-PAGE and probed by a rabbit polyclonal TAP antibody (Invitrogen; CAB1001). A gel slice that does not contain immunoblotting signals was stained by Simple Blue Safe (Invitrogen) as a loading control.

### Affinity purification mass spectrometry

#### Cell preparation and protein extraction

GFP-tagged Mfg1 or GFP-tagged Eno1 (negative control) strains were grown in YPD overnight at 30°C. Cells were diluted to an OD_600_ of 0.1 in 500 mL of YPD (2 x 500 mL for untreated cells) and grown to an OD of 0.6–0.8 at 30°C, at which point untreated cells were harvested at 4000 rpm for 20 minutes. Cells to be treated with serum were washed with PBS and grown for an additional 1 hour or 3 hours in the presence of 10% heat-inactivated new-born calf serum (Gibco), at which point cells were harvested as before.

To extract proteins, lysis buffer (50 mM HEPES-Na (pH 7.5), 150 mM NaCl, 5 mM EDTA, 5 mM DTT, 0.1% NP-40, 1x ROCHE protease inhibitors) was added 1:1 to the cell pellets (g:mL), and vortexed at 4°C with an equivalent volume of glass beads (0.5 mm) for 4 x 1 minute, with 1 minute on ice in between. A needle (27 ½) was used to poke a hole in the tube, which was then spun at 1000 rpm for 1–2 minutes at 4°C. This lysate was sonicated (Q SONICA, probe CL-18, power 30%) at 4°C for 3 x 10 seconds with 3 seconds between pulses. Twenty-five units of Benzonase Nuclease (EMD Millipore) was added and samples were rotated at 4°C for 30 minutes, before centrifugation at 14,000 rpm for 20 minutes at 4°C.

GFP-tagged proteins were affinity-purified using the GFP-Trap resin (Chromotek). Resin was equilibrated three times with 1 mL of lysis buffer, and 25 μL of resin was used per 1 L of culture. Extracted proteins were added to resin and incubated at 4°C for 2 hours with rotation. Beads were washed with 1 mL of lysis buffer, followed by 1 mL of wash buffer (20 mM Tris-Hcl (pH 8.0), 2 mM CaCl_2_). Beads were then washed with 20 mM Tris-HCl (pH 8.0), and incubated with 1 μg of trypsin (Sigma) at 37°C for 4 hours with rotation. Beads were removed magnetically and 0.5 μg of trypsin was added for incubation at 37°C without rotation overnight. Formic acid was then added to a final concentration of 0.2%, and dried. Peptides were stored at -80°C until analysis.

#### MS/MS

Affinity purified and digested peptides were analyzed using nano-HPLC (High-performance liquid chromatography) coupled to MS on the AB SCIEX 5600 TripleTOF in data-dependent acquisition (DDA) mode. Nano-spray emitters were generated from fused silica capillary tubing, with 75 μm internal diameter, 365 μm outer diameter and 5–8 μm tip opening, using a laser puller (Sutter Instrument Co., model P-2000, with parameters set as heat: 280, FIL = 0, VEL = 18, DEL = 2000). Nano-spray emitters were packed with C18 reversed-phase material (Reprosil-Pur 120 C18-AQ, 3μm) resuspended in methanol using a pressure injection cell. 5 μl of each sample was directly loaded at 400 nl/min for 10 min onto a 75 μmx10 cm nano-spray emitter. Peptides were eluted from the column with an acetonitrile gradient generated by an Eksigent NanoLC-Ultra 1D plus, and analyzed on a TripleTOF 5600 instrument (AB SCIEX, Concord, Ontario, Canada). The gradient was delivered at 200 nl/min from 2% acetonitrile with 0.1% formic acid to 35% acetonitrile with 0.1% formic acid using a linear gradient of 90 min. This was followed by a 5 min wash with 80% acetonitrile with 0.1% formic acid, return to 2% acetonitrile over 5 min and equilibration for another 15min at 2% acetonitrile with 0.1% formic acid. The total DDA protocol is 120 min. The first DDA scan had an accumulation time of 250ms within a mass range of 400–1250 Da. This was followed by up to 20 MS/MS scans, with accumulation time of 100 ms for each MS/MS scan. Each candidate ion was required to have a charge state from 2–4 and a minimum threshold of 250 counts per second, isolated using a window of 50 mDa. Previously analyzed candidate ions were dynamically excluded for 15 seconds.

#### AP-MS data analysis and visualization

Mass spectrometry data generated were stored, searched and analyzed using ProHits laboratory information management system (LIMS) platform [70]. Within ProHits, WIFF files were converted to an MGF format using the WIFF2MGF converter and to an mzML format using ProteoWizard (V3.0.10702) and the AB SCIEX MS Data Converter (V1.3 beta). The data was then searched using Mascot (V2.3.02) [71] and Comet (V2016.01 rev.2) [72]. The spectra were searched with the *C*. *albicans* sequences in the RefSeq database (txid5476[Organism:exp]) acquired from NCBI, supplemented with “common contaminants” from the Global Proteome Machine (GPM; ftp://ftp.thegpm.org/fasta/cRAP/crap.fasta) and forward&reverse sequences (labeled “DECOY”) for a total of 30,018 entries. Database parameters were set to search for tryptic cleavages, allowing up to 2 missed cleavages sites per peptide with a mass tolerance of 35ppm for precursors with charges of 2+ to 4+ and a tolerance of 0.15 amu for fragment ions. Variable modifications were selected for deamidated asparagine and glutamine and oxidized methionine. Results from each search engine were analyzed through TPP (the Trans-Proteomic Pipeline, v.4.7 POLAR VORTEX rev 1) via the iProphet pipeline [73].

SAINTexpress version 3.6.1 was used as a statistical tool to calculate the probability of potential protein-protein interactions from background contaminants using default parameters [74]. SAINT analysis was performed using two biological replicates per bait. Six negative control experiments were conducted using GFP-Eno1 as bait. Eno1-GFP cells were grown and treated identically to the GFP-Mfg1 cells. Controls were compressed to 2 samples and two unique peptide ions and a minimum iProphet probability of 0.95 were required for protein identification. SAINT probabilities were calculated independently for each sample, averaged (AvgP) across biological replicates and reported as the final SAINT score. Only SAINT scores with a false discovery rate (FDR) ≤ 1% were considered high-confidence protein interactions. Dot plots were generated using the “Dot plot generator” tool in ProHits-viz [75] using SAINTexpress file generated from ProHits. Parameters set in the tool were: primary FDR cutoff = 0.01, secondary FDR cutoff = 0.05 and maximum spectral count = 50. Hierarchical clustering was performed using Pearson correlation and average linkage.

All MS files used in this study were also deposited as a complete submission to the MassIVE respository (https://massive.ucsd.edu/ProteoSAFe/static/massive.jsp) and assigned the accession number MSV000083218. The ProteomeXchange accession is PXD012004.

### Quantitative reverse transcription-PCR (qRT-PCR)

Cells were grown overnight in YPD at 30°C, diluted to an OD_600_ of 0.1, and grown at 30°C to mid-log phase. To verify overexpression of various genes in the *mfg1Δ/mfg1Δ* background, cells were subcultured to an OD_600_ of 0.1 in YPD in the presence of 10% serum and grown at 37°C to mid-log phase. To examine expression of *TPK2*, cells were grown as above, but subcultured to OD_600_ of 0.1 and grown at 30°C or 34°C to mid-log phase. Cultures were pelleted and frozen at -80°C. RNA extraction, complementary DNA synthesis and PCR were performed as previously described [18]. Reactions were performed in triplicate, for two biological replicates and data were analyzed using the BioRad CFX Manager 3.1. Transcript levels were examined using the primers, oLC1988/oLC1989 (*TEF1*), oLC2285/oLC2286 (*ACT1*), oLC3796/oLC751 (*HWP1*), oLC5038/oLC5320 (*MFG1*), oLC5040/oLC5323 (*MSS11*), oLC5036/oLC5322 (*FLO8*), oLC4839/oLC4840 (*SUR7*), oLC4831/oLC4832 (*HSP21*), oLC2637/oLC2638 (*ROB1*), oLC5699/oLC829 (*TPK2*), oLC6472/oLC6473 (*PMA1*), oLC6476/oLC6477 (*RIP1*), oLC6738/oLC6739 (orf19.3897), oLC6736/oLC6737 (*PGA26*), oLC1456/oLC1457 (*IHD1*), oLC6718/oLC6719 (*TEC1*), and oLC2635/oLC2636 (*BRG1*). All oligonucleotide sequences are listed in S7 Table.

### Microscopy and imaging

Pseudohyphal growth of diploid *S*. *cerevisiae* cells was assayed on SLAD solid medium supplemented with 15 μM leucine and 10 μM histidine as previously described [26,62]. Plates were incubated at 30°C for 11 days. Images of single colonies were taken on a Zeiss Axio Observer.Z1 (Carl Zeiss). Invasive growth of haploid *S*. *cerevisiae* was assayed by spotting equal cell dilutions on YPD plates with 2% agar. Plates were incubated at 30°C for 4 days before washing with water to remove non-invasive cells. Images were taken before and after washing with a Canon Power Shot A610. To image *C*. *albicans*, cells were grown overnight in YPD at 30°C. Cultures were diluted to an OD_600_ of 0.1 and grown in the indicated conditions. Cells were imaged using differential interference contrast (DIC) microscopy using a Zeiss Axio Imager.MI (Carl Zeiss). All images are representative of multiple fields of view from at least biological duplicate experiments.

For nuclear staining and microscopic analysis of cell morphology, cells were harvested, washed once with PBS and resuspended in 1 mL PBS containing 5 μg/mL Hoechst 33342. Following an incubation of 15 min in the dark at room temperature, cells were spun down and resuspended in 50 μL of PBS and imaged. Imaging was performed on a Zeiss Imager M1 upright microscope at 40X magnification on the green fluorescent protein (GFP) channel for GFP tagged proteins, the DAPI (4’,6-diamidino-2-phenylindole) channel for nuclei stained with Hoechst 33342 and the DIC channel. At least three fields were imaged for each strain, in at least two biological replicates.

## Supporting information

S1 Text Details of strain and plasmid construction. (DOCX)

S1 Fig Complementation of the *flo8Δ/flo8Δ* mutant with a wild-type allele of *FLO8* restores its ability to filament. Cells were grown in the conditions indicated and imaged after 3.5 hours. Scale bar is 20 μm.
(TIFF)

S2 Fig Deletion of *MSS11* does not block filamentous growth in a variety of inducing cues. Two independently generated *mss11Δ/mss11Δ* mutants, M1 from Fig 1 and a second mutant M2, show no defect in filamentation in response to any cue tested. Cells were grown in the conditions indicated, and were imaged after 3.5 hours, except for those grown in the presence of GdA, which were imaged after 24 hours. Scale bar is 20 μm.
(TIFF)

S3 Fig Replacing the native promoter of *FLO8* or *MSS11* with a tetracycline-repressible promoter in an *mfg1Δ/mfg1Δ* mutant results in overexpression of these regulators. Quantification of overexpression of *FLO8* or *MSS11* in the *mfg1****Δ****/mfg1****Δ*** mutant by qRT-PCR. Cells were grown in YPD at 30°C for 3 hours. Transcript levels were normalized to *ACT1* and *TEF1* and error bars represent standard error of technical triplicates. Assays were performed in biological duplicate. Asterisks indicate P< 0.0001 (***) relative to parental strain (two-tailed unpaired t-test).
(TIFF)

S4 Fig Flo8 and Mfg1 localize to the nucleus, and tagged proteins are functional. **A)** Cells expressing Mfg1-GFP or Flo8-GFP were grown in either YPD at 30°C or YPD with 10% serum at 37°C for 1 hour. Cells were treated with Hoechst 33342 dye to monitor nuclear localization. **B)** Cells expressing Flo8-TAP or Mfg1-TAP were grown in YPD with 10% serum at 37°C for 6 hours. Scale bar is 20 μm. **C)** Mfg1-GFP cells were grown in YPD at 30°C or in YPD with 10% serum at 37°C for 2 hours. Scale bar is 20 μm.
(TIFF)

S5 Fig ChIP-chip and transcriptional analyses reveal a core set of genes bound by both Flo8 and Mfg1, but also many condition-specific and temporal differences. **A)** Cells for ChIP-chip were grown in YPD at 30°C until mid-log phase (basal), and then treated for 1 hour or 3 hours in 10% serum at 37°C. Genes were clustered using Cluster Gene 3.0 and visualized using JavaTreeView, where green represents an increased binding intensity. **B)** ChIP-chip analysis reveals a core set of genes bound by both Flo8 and Mfg1 under basal (untreated 30°C) and filament-inducing (serum 37°C) conditions (top Venn diagram). Our analysis also identified many unique targets under all environmental conditions, as well as substantial changes in promoter binding upon exposure to serum (bottom Venn diagrams). **C)** Wild-type, *flo8****Δ****/flo8****Δ***, *mfg1****Δ****/mfg1****Δ***, or *flo8****Δ****/flo8****Δ*** *mfg1****Δ****/mfg1****Δ*** strains were grown as in A) for transcriptional analysis by microarray. Heat map was generated as in A. Plotted are the log_2_ fold-change in expression of the mutant strain relative to wild type. **D)** Microarray analysis reveals that Flo8 and Mfg1 have distinct effects on gene expression (DEG: differentially expressed gene) and identifies temporal transcriptional changes that occur in response to serum.
(TIFF)

S6 Fig Overexpression or deletion of specific target genes downstream of Mfg1 do not restore the ability of an *mfg1Δ/mfg1Δ* mutant to filament. **A)** Select genes both bound and transcriptionally regulated by Mfg1 upon exposure to serum were overexpressed (positive regulators of filamentation), or deleted (negative regulator of filamentation) in an *mfg1****Δ****/mfg1****Δ*** mutant. Cells were grown in YPD at 30°C, or in YPD with 10% serum at 37°C for 5 hours. Scale bar is 20 μm. **B)** Quantification of overexpression of target genes by qRT-PCR. Overexpression of each target open reading frame was achieved by replacing the native promoter of one or both alleles for each gene with a tetracycline-repressible promoter, *tetO*. Cells were grown in YPD in the presence of 10% serum at 37°C for 6 hours. Transcript levels were monitored using qRT-PCR and normalized to *ACT1* and *TEF1*. Error bars represent standard error of technical triplicates. Assays were performed in biological duplicate. Asterisks indicate P < 0.0001 (***), relative to the parental strain (one-way ANOVA, Bonferroni Multiple Comparison Test). **C)** Replacing both native *TEC1* promoters with the strong *tetO* promoter results in increased *TEC1* expression. Cells were grown in YPD at 30°C for 4 hours. Transcript levels were monitored using qRT-PCR and normalized to *ACT1* and *TEF1*. Error bars represent standard error of technical triplicates. Assays were performed in biological duplicate. Asterisks indicate P < 0.0001 (***), relative to the parental strain (one-way ANOVA, Bonferroni Multiple Comparison Test).
(TIFF)

S7 Fig Deletion of either *FLO8* or *MFG1* does not affect transcript levels of the other regulator, but Mfg1 protein levels are reduced in the absence of Flo8. **A)** Deletion of *FLO8* or *MFG1* does not alter the expression of the other regulator. Cells were grown in YPD at 30°C for 4 hours. Transcript levels were monitored using qRT-PCR and normalized to *ACT1* and *TEF1*. Error bars represent standard error of technical triplicates. Assays were performed in biological duplicate. Asterisks indicate P < 0.0001 (***), relative to the wild type (two-tailed unpaired t-test). **B)** Protein levels of Mfg1-TAP and Flo8-TAP in wild-type cells or cells lacking the other regulator were monitored with immunoblotting. Cells were grown in YPD at 30°C for 5 hours. Staining with Simple Blue Safe (SBS) was used as a loading control. ‘Loading’ indicates relative amount of lysate loaded.
(TIFF)

S8 Fig Mfg1 and Flo8 bind to the promoter of *BRG1*, whose expression increases upon *FLO8* overexpression. **A)** Binding of Flo8-TAP and Mfg1-TAP to the promoter of *BRG1* was assessed using ChIP-qPCR. Shown is the fold-enrichment over the untagged parental strain, which is set at 1. Asterisks indicate P < 0.01 (**) or P < 0.001 (***), relative to the wild-type parent in the respective condition (two-tailed unpaired t-test). Error bars represent the standard deviation of technical triplicates. Assays were performed in biological duplicate. **B**) *BRG1* expression is decreased in the absence of Flo8 or Mfg1. Cells were grown in YPD at 30°C for 3.5 hours, and then transferred to 37°C YPD with 10% serum for 1 hour. Transcript levels were monitored using qRT-PCR and normalized to *PMA1* and *RIP1*. Error bars represent standard error of technical triplicates. Assays were performed in biological duplicate. Asterisks indicate P < 0.001 (***) or P < 0.05 (*), relative to the wild-type strain in each respective condition (two-way ANOVA, Bonferroni Multiple Comparison Test). **C)** *BRG1* expression is increased in strains overexpressing *FLO8*. Cells were grown in YPD at 30°C for 4.5 hours. Transcript levels were monitored using qRT-PCR and normalized to *PMA1* and *RIP1*. Error bars represent standard error of technical triplicates. Assays were performed in biological duplicate. Asterisks indicate P < 0.0001 (***), relative to the respective parental strain, or as indicated (one-way ANOVA, Bonferroni Multiple Comparison Test).
(TIFF)

S9 Fig Expression of *FLO8* is increased in evolved lineages with increased *FLO8* copy number, and mutants revert to yeast form growth after 24 hours in serum. **A)** Evolved mutants revert to yeast form after 24 hours of growth in serum. Cells were grown in YPD at 30°C or in YPD with 10% serum at 37°C for 24 hours. Scale bar represents 20 μm. **B)** Expression of *FLO8* is increased in evolved lineages with increased *FLO8* copy number. Cells were grown in YPD at 30°C for 3.5 hours. Transcript levels were monitored using qRT-PCR and normalized to *PMA1* and *RIP1*. Error bars represent standard error of technical triplicates. Assays were performed in biological duplicate. Asterisks indicate P < 0.001 (***) and P < 0.01 (**), relative to the parent (two-tailed unpaired t-test). **C)** Expression of *BRG1* and *TEC1* are increased in the evolved lineage Evo #A2 in the presence of 10% serum at 37°C. Cells were grown in YPD at 30°C for 3.5 hours, before being transferred to 37°C YPD with 10% serum for 1 hour. Transcript levels were monitored using qRT-PCR and normalized to *PMA1* and *RIP1*. Error bars represent standard error of technical triplicates. Assays were performed in biological duplicate. Asterisks indicate P < 0.05 (*), relative to the parental strain (two-tailed unpaired t-test). **D)** NAT-resistant colonies were not obtained when the *flo8****Δ****/flo8****Δ*** mutant or the *mfg1****Δ****/mfg1****Δ*** *flo8****Δ****/flo8****Δ*** *mss11****Δ****/mss11****Δ*** triple mutant were plated on 10% serum and 250 μg/mL of NAT. 4 x10^7^ cells were plated in triplicate, and incubated for 72 hours.
(TIFF)

S10 Fig *FLO8* at the *HIS1* locus is functional, and *FLO8* expression increases with *FLO8* copy number. **A)** Expression of Flo8-repressed genes does not increase in the strain in which *FLO8* is at the *HIS1* locus on chromosome 5. Cells were grown in YPD at 30°C for 4 hours or in YPD with 10% serum at 37°C for 3 hours. Transcript levels were monitored using qRT-PCR and normalized to *PMA1* and *TEF1*. Error bars represent standard error of technical triplicates. Assays were performed in biological duplicate. Asterisks indicate P < 0.001 (**), relative to the wild type (two-way ANOVA, Bonferroni correction). **B)** Most filament-capable *mfg1****Δ****/mfg1****Δ*** mutants with *FLO8* at a non-native locus revert to yeast form growth after 24 hours in serum. Cells were grown in YPD at 30°C or in YPD with 10% serum at 37°C for 24 hours. Mutants Evo #C1-1 and Evo #C1-2 arose from the same overnight culture but showed distinct phenotypes in terms of their ability to filament at a later time point. Mutants Evo #C1, C2, C3, and C4 are all independently generated. Scale bar is 20 μm. **C)** Expression of *FLO8* is most increased in the evolved lineage with increased *FLO8* copy number. Cells were grown in YPD at 30°C for 3.5 hours. Transcript levels were monitored using qRT-PCR and normalized to *PMA1* and *TEF1*. Error bars represent standard error of technical triplicates. Assays were performed in biological duplicate. Asterisks indicate P < 0.001 (***), P < 0.01 (**), or P < 0.05 (*) relative to the parent (two-tailed unpaired t-test). **D)** A model depicting the roles of Mfg1 and Flo8 in governing expression of key regulators of filamentation. Mfg1 and Flo8 are both required for optimal binding to the promoters of *BRG1* and *TEC1*, however, overexpression of *FLO8* in the absence of Mfg1 is sufficient to drive filamentation and expression of *BRG1* and *TEC1*.
(TIFF)

S1 Table ChIP-chip analysis. List of targets bound by Mfg1 or Flo8 under basal conditions (30°C) or filament-inducing conditions (10% serum for one hour or 3 hours, 37°C).
(XLSX)

S2 Table Microarray analysis. List of targets whose expression is altered in the *mfg1****Δ****/mfg1****Δ***, *flo8****Δ****/flo8****Δ***, or *mfg1****Δ****/mfg1****Δ*** *flo8****Δ****/flo8****Δ*** double mutant, under basal conditions (30°C) or filament-inducing conditions (10% serum for one hour or 3 hours, 37°C).
(XLSX)

S3 Table Genes bound and regulated by Mfg1 or Flo8. List of targets bound by Mfg1 or Flo8 and whose expression is altered in the *mfg1****Δ****/mfg1****Δ*** or *flo8****Δ****/flo8****Δ*** mutants respectively.
(XLSX)

S4 Table AP-MS MassIVE output files. *MassIVE Table 1*: Sample description table. Baits are Mfg1-GFP grown in YPD at 30°C (MFG1_untreat), or in the presence of 10% serum at 37°C for 1 hour (MFG1_1) or for 3 hours (MFG1_3). Eno1-GFP was used as the negative control, grown in the same conditions. *MassIVE Table 2*: Protein identification evidence. *MassIVE Table 3*: MassIVE SAINTexpress v.3.6.1 output. Prey Accession is the NCBI protein accession number; Prey Gene is as per NCBI Entrez Gene. Spectral counts for the prey (column D, separated by "I" delimiter; column E, summed, column F, averaged), number of replicates performed (column G), spectral counts for the prey across all negative controls (column H), Averaged probability across replicates (column I), maximal probability (column J), log Odds score (column K), Fold Change (counts in the purification divided by counts in the controls plus small factor to prevent division by 0; column L) and Bayesian FDR (column M) are listed for each bait-prey relationship and are directly from the SAINTexpress output. Columns N-P are the unique prey peptides as calculated through ProHits. Column Q is the prey protein length and column R is the UniProt accession number. The values for Mfg1, Flo8, and Mss11 are highlighted in yellow.
(XLS)

S5 Table Strains used in this study, including SNPs in the evolved lineages identified by whole genome sequencing. (XLSX)

S6 Table Plasmids used in this study. (XLSX)

S7 Table Oligonucleotides used in this study. (XLSX)
