# Supplementary material for: Functional divergence of a global regulatory complex governing fungal filamentation
Source: PLoS Genet. 2019 Jan 7;15(1):e1007901. doi: 10.1371/journal.pgen.1007901 (PMC6336345; doi:10.1371/journal.pgen.1007901)
Supplement: S1 Text — (DOCX) [file pgen.1007901.s001.docx]

**Strain construction:**

**CaLC1933:** The TAP-HIS1 construct was amplified from pLC572 using oLC1956/oLC1957 containing homology to *FLO8*. The PCR product was transformed into CaLC239. HIS prototrophs were PCR tested for proper integration using oLC1634/oLC1958 and oLC1645/oLC1959.

**CaLC2275:** The *MFG1* knockout construct was PCR-amplified from pLC49 using oLC1901/oLC1892 containing homology to *MFG1*. The PCR product was transformed into CaLC239. NAT-resistant transformants were PCR tested for proper integration of the construct using primers oLC1902/oLC275 and oLC274/oLC1894. The *SAP2* promoter was induced to drive expression of the FLP recombinase to excise the NAT marker cassette.

**CaLC2284:** The TAP-HIS1 construct was amplified from pLC572 using oLC1953/oLC1954 and transformed into CaLC2275. HIS prototrophs were PCR tested for proper integration using oLC1634/oLC1955 and oLC1645/oLC1894. Absence of the wild-type untagged allele was confirmed with oLC1920/oLC1894 and presence of the deleted allele was confirmed with oLC1902/oLC1894.

**CaLC2758:** To tag Mfg1 with a C-terminal GFP tag, GFP-NAT was amplified from pLC389 with oLC2639 and oLC2640, and the construct was transformed into CaLC239. Proper upstream integration was verified using oLC2641 and oLC600. Proper downstream integration was verified using oLC2642 and oLC601.

**CaLC2885:** The *FLO8* knockout construct was PCR-amplified from pLC49 using oLC2818/oLC2819 containing sequence homologous to upstream and downstream regions of *FLO8*, and transformed into the wild type CaLC239. NAT-resistant transformants were PCR tested for proper integration of the construct using primers oLC275/oLC2820 and oLC274/oLC2821. The *SAP2* promoter was induced to drive expression of the FLP recombinase to excise the NAT marker cassette.

**CaLC2938:** The *MSS11* knockout construct was PCR-amplified from pLC49 using primer pair oLC2881/2882, containing sequence homologous to upstream and downstream regions of *MSS11*, and transformed into the wild type CaLC239. NAT-resistant transformants were PCR tested for proper integration of the construct using primer pairs oLC2883/oLC275 and oLC274/oLC2885. The *SAP2* promoter was induced to drive expression of the FLP recombinase to excise the NAT marker cassette.

**CaLC2945:** To tag Mfg1 with a C-terminal TAP tag in a *flo8Δ/flo8Δ* background, the TAP construct was amplified from pLC572 with oLC1953 and oLC1954, and transformed into CaLC2897. Proper upstream integration was verified using oLC1634 and oLC1955, and proper downstream integration was verified using oLC1645 and oLC1894.

**CaLC3092:** To make a *flo8Δ/ flo8Δ mfg1Δ/mfg1Δ* double null, the NAT cassette was PCR amplified from pLC49 using primers oLC1892 and oLC1901 and transformed into CaLC2897. NAT-resistant transformants were PCR tested for proper integration with oLC1902/oLC275 and oLC274/oLC1894. The *SAP2* promoter was induced to drive expression of FLP recombinase to excise the NAT marker cassette. The second allele was deleted in the same manner. Absence of a wild-type allele was confirmed with primers oLC1902/oLC1894 and oLC1920/oLC1921.

**CaLC3094:** The *MSS11* knockout construct was PCR-amplified from pLC49 using primer pair oLC2881/2882, containing sequence homologous to upstream and downstream regions of *MSS11*, and transformed into the wild type CaLC239. NAT-resistant transformants were PCR tested for proper integration of the construct using primer pairs oLC2883/oLC275 and oLC274/oLC2885. The *SAP2* promoter was induced to drive expression of the FLP recombinase to excise the NAT marker cassette. The second *MSS11* allele was deleted in the same manner. Absence of a wild-type allele of *MSS11* was verified using oLC2883/oLC2885 and oLC2883/2884.

**CaLC3861**: To tag the NAT cassette with a *HIS* marker, the *HIS* cassette was amplified from pLC575 [1] with oLC3847 and oLC3848 and transformed into CaLC3844. *HIS* prototrophic transformants were PCR tested for proper integration with oLC3849/oLC2029 and oLC1645/oLC3850 for downstream integration.

**CaLC3900:** To drive the expression of NAT with *HWP1* promoter, the NAT-*HIS* cassette was amplified from the genomic DNA of CaLC3861 with oLC3875 and oLC3876 (~5 kb). The PCR product was cleaned up by spin column purification and used as a template for amplification with oLC3851 and oLC3852 (2357 bp). The *HWP1p*-NAT-*HIS* construct was transformed into CaLC239. *HIS* prototrophic transformants were PCR tested for proper integration with oLC3887/oLC2029 and oLC1645/oLC3855.

**CaLC4449:** To tag Eno1 with a C-terminal GFP tag, GFP-NAT was amplified from pLC389 with oLC596 and oLC597 and the construct was transformed into CaLC239. Proper upstream integration was verified using oLC598 and oLC600, and proper downstream integration was verified using oLC599 and oLC601.

**CaLC4651:** To overexpress *SUR7* in the *mfg1Δ/mfg1Δ* background, the NAT cassette was amplified from pLC605 using primers oLC4635/oLC4636 and transformed into CaLC1909. NAT-resistant transformants were PCR tested for proper integration with oLC534/oLC4638 and oLC300/oLC4637. The *SAP2* promoter was induced to drive expression of FLP recombinase to excise the NAT marker cassette.

**CaLC4653:** To overexpress *ROB1* in the *mfg1Δ/mfg1Δ* background, the NAT cassette was amplified from pLC605 using primers oLC4643/oLC4644 and transformed into CaLC1909. NAT-resistant transformants were PCR tested for proper integration with oLC534/oLC4645 and oLC300/oLC4646. The *SAP2* promoter was induced to drive expression of FLP recombinase to excise the NAT marker cassette.

**CaLC4701:** To overexpress *HSP21* in the *mfg1Δ/mfg1Δ* background, the NAT cassette was amplified from pLC605 using primers oLC4639/oLC4640 and transformed into CaLC1909. NAT-resistant transformants were PCR tested for proper integration with oLC534/oLC4642 and oLC300/oLC4641. The *SAP2* promoter was induced to drive expression of FLP recombinase to excise the NAT marker cassette.

**CaLC4765**: To delete *CUP9* from the *mfg1Δ/mfg1Δ* background, the NAT cassette was PCR amplified from pLC49 using primers oLC3669 and oLC3670 and transformed into CaLC1909. Proper upstream integration was verified using oLC275/oLC4817 and proper downstream integration was verified using oLC274/oLC4818. The *SAP2* promoter was induced to drive expression of the FLP recombinase to excise the NAT marker cassette. The second allele was deleted in the same manner. Absence of a wild-type allele was verified with oLC4817/oLC4818.

**CaLC4809**: To overexpress *MSS11*, the NAT cassette was amplified from pLC605 with primers oLC4973 and oLC4974 and transformed into CaLC239. NAT-resistant transformants were PCR tested for proper integration with primers oLC534/oLC4975 and oLC300/oLC4969. The *SAP2* promoter was induced to drive expression of FLP recombinase to excise the NAT marker cassette.

**CaLC4832**: To overexpress *FLO8*, the NAT cassette was amplified from pLC605 with primers oLC4971 and oLC4972 and transformed into CaLC239. NAT-resistant transformants were PCR tested for proper integration with primers oLC534/oLC2820 and oLC300/oLC4962. The *SAP2* promoter was induced to drive expression of FLP recombinase to excise the NAT marker cassette.

**CaLC4920:** To drive the expression of NAT with *HWP1* promoter in an *mfg1Δ/mfg1Δ* background, the NAT-*HIS* cassette was amplified from the genomic DNA of CaLC3861 with oLC3875 and oLC3876 (~5 kb). The PCR product was cleaned up by spin column purification and used as a template for amplification with oLC3851 and oLC3852 (2357 bp). The *HWP1p*-NAT-*HIS* construct was transformed into CaLC1909. *HIS* prototrophic transformants were PCR tested for proper integration with oLC3854/oLC3887 and oLC242/oLC3855.

**CaLC4934**: To overexpress *FLO8* in an *mfg1∆/∆* background*,* the NAT cassette was amplified from pLC605 with primers oLC4971 and oLC4972 and transformed into CaLC1909. NAT-resistant transformants were PCR tested for proper integration with primers oLC2820/oLC534 and oLC300/oLC4967. The *SAP2* promoter was induced to drive expression of FLP recombinase to excise the NAT marker cassette.

**CaLC4943:** To select for mutants in the *mfg1Δ/mfg1Δ* background that are able to express the NAT cassette, 4x10^7^ cells of CaLC4920 (overnight #1) were plated on YPD+10% serum +250 μg/mL NAT. NAT resistant colonies appeared after 2 days at 37°C.

**CaLC4972:** To select for mutants in the *mfg1Δ/mfg1Δ* background that are able to express the NAT cassette, 4x10^7^ cells of CaLC4920 (overnight #2) were plated on YPD+10% serum +250 μg/mL NAT. NAT resistant colonies appeared after 2 days at 37°C.

**CaLC4973:** To select for mutants in the *mfg1Δ/mfg1Δ* background that are able to express the NAT cassette, 4x10^7^ cells of CaLC4920 (overnight #3) were plated on YPD+10% serum +250 μg/mL NAT. NAT resistant colonies appeared after 2 days at 37°C.

**ScLC4979:** To complement *S. cerevisiae mfg1Δ/Δ,* ScLC4774 was transformed with pLC984. URA prototrophs were PCR tested with primers oLC1920/oLC1921 to confirm presence of the plasmid.

**ScLC4981**: To create a marker-matched *flo81Δ/Δ* diploid control, ScLC4773 was transformed with pLC155. URA prototrophs were PCR tested with primers oLC1868/oLC1869 to confirm presence of the plasmid.

**ScLC4985:** To create a marker-matched *S. cerevisiae* *mfg1Δ/Δ* control, ScLC4774 was transformed with pLC155. URA prototrophs were PCR tested with primers oLC1868/oLC1869 to confirm presence of the plasmid.

**ScLC4987:** To create a marker-matched wild-type diploid control, ScLC4777 was transformed with pLC155. URA prototrophs were PCR tested with primers oLC1868/oLC1869 to confirm presence of the plasmid.

**ScLC5020**: To complement *S. cerevisiae flo8Δ/Δ,* ScLC4773 was transformed with pLC987. URA prototrophs were PCR tested with primers oLC5321/oLC5322 to confirm presence of the plasmid.

**ScLC5024:** To create a marker-matched *S. cerevisiae* *mfg1Δ* control, ScLC4771 was transformed with pLC155. URA prototrophs were PCR tested with primers oLC1868/oLC1869 to confirm presence of the plasmid.

**ScLC5026:** To create a marker-matched wild-type haploid control, ScLC4775 was transformed with pLC155. URA prototrophs were PCR tested with primers oLC1868/oLC1869 to confirm presence of the plasmid.

**ScLC5030**: To complement *S. cerevisiae* *mfg1Δ,* ScLC4771 was transformed with pLC984. URA prototrophs were PCR tested with primers oLC1920/oLC1921 to confirm presence of the plasmid.

**CaLC5043:** To make an *mfg1Δ/mfg1Δ flo8Δ/flo8Δ mss11Δ/mss11Δ* triple null mutant, pLC1008 (containing the CRISPR guide gRNA to *MSS11*) was digested with KpnI and SacI and transformed into CaLC3092 along with the repair DNA (760bp product PCR-amplified from CaLC2938 gDNA with oLC4975/oLC2885). Integration of the repair was verified with oLC4975/oLC2885. The *SAP2* promoter was induced to drive expression of the FLP recombinase to excise the NAT marker cassette. Absence of a wild-type allele of *MSS11* was verified using oLC2883/oLC2884. Absence of *FLO8* alleles (oLC2820/2846) and absence of *MFG1* alleles (oLC1920/1921) were also verified.

**CaLC5048:** To drive the expression of NAT with *HWP1* promoter in an *mfg1Δ/mfg1Δ f1o8Δ/flo8Δ mss11Δ/mss11Δ* background, the NAT-*HIS* cassette was amplified from the genomic DNA of CaLC4920 with primers oLC3851/oLC3852 and transformed into CaLC5043. ARG prototrophs were verified for proper integration with oLC3854/3887 and oLC242/3855.

**CaLC5056**: To overexpress *MFG1*, the NAT cassette was amplified from pLC605 with primers oLC1891 and oLC4970 and transformed into CaLC239. NAT-resistant transformants were PCR tested for proper integration with primers oLC300/oLC4968 and oLC300/oLC301 and oLC300/oLC5318. The *SAP2* promoter was induced to drive expression of FLP recombinase to excise the NAT marker cassette.

**CaLC5075**: To create an independent *MSS11* null, pLC1008 (containing the CRISPR guide gRNA to *MSS11*) was digested with KpnI and SacI and transformed into CaLC239 along with the repair DNA (760bp product PCR-amplified from CaLC2938 gDNA with oLC4975/oLC2885). Integration of the repair was verified with oLC4975/oLC2885. The *SAP2* promoter was induced to drive expression of the FLP recombinase to excise the NAT marker cassette. Absence of a wild-type allele of *MSS11* was verified using oLC2883/oLC2884 and oLC5040/oLC5325.

**CaLC5131:** Fusion PCR was performed to knock out an allele of *FLO8* with *CdARG4*. Upstream homology was amplified with oLC4976/oLC5617 from genomic DNA of CaLC155 (SC5314). Downstream homology was amplified with oLC5618/oLC2821 from genomic DNA of CaLC155 (SC5314). The *CdARG4* cassette was amplified from pLC45 with oLC5616/oLC5619. PCR fusion of all three pieces was performed with oLC4976 and oLC2821. This product was transformed into CaLC4920. ARG prototrophs were verified for proper integration using primers oLC921/oLC5549 and oLC5548/oLC1959.

**CaLC5133:** Fusion PCR was performed to knock out an allele of *FLO8* with *CdARG4*. Upstream homology was amplified with oLC4976/oLC5617 from genomic DNA of CaLC155 (SC5314). Downstream homology was amplified with oLC5618/oLC2821 from genomic DNA of CaLC155 (SC5314). The *CdARG4* cassette was amplified from pLC45 with oLC5616/oLC5619. PCR fusion of all three pieces was performed with oLC4976 and oLC2821. This product was transformed into CaLC4972. ARG prototrophs were verified for proper integration using primers oLC921/oLC5549 and oLC5548/oLC1959.

**CaLC5143:** To overexpress *MSS11* in an *mfg1∆/∆* background*,* the NAT cassette was amplified from pLC605 with primers oLC4974 and oLC4973 and transformed into CaLC1909. NAT-resistant transformants were PCR tested for proper integration with primers oLC534/oLC4975 and oLC300/oLC4969. The *SAP2* promoter was induced to drive expression of FLP recombinase to excise the NAT marker cassette.

**CaLC5154**: To overexpress *TPK2*, the NAT cassette was amplified from pLC605 with primers oLC5697/oLC5696 and transformed into CaLC239. NAT-resistant transformants were PCR tested for proper integration with primers oLC5698/oLC534 and oLC5699/oLC4714. The *SAP2* promoter was induced to drive expression of the FLP recombinase to excise the NAT marker cassette.

**CaLC5173:** To create a marker-matched parent, the *CdARG4* cassette was amplified from pLC45 with oLC5753/oLC5754 and transformed into CaLC4920. ARG prototrophs were PCR tested for proper integration with oLC5549/oLC5755 and oLC5548/oLC5756. Presence of *CdARG4* confirmed with oLC5927/oLC5928.

**CaLC5175:** To create a marker-matched parent, the *CdARG4* cassette was amplified from pLC45 with oLC5753/oLC5754 and transformed into CaLC4972. ARG prototrophs were PCR tested for proper integration with oLC5549/oLC5755 and oLC5548/oLC5756. Presence of *CdARG4* confirmed with oLC5927/oLC5928.

**CaLC5185:** The *FLO8* ORF +/-1000bp was amplified from CaLC155 (SC5314) genomic DNA with oLC5710/oLC5708. This PCR product was amplified with homology to *HIS1* using primers oLC5759/oLC5752. This product is the repair. Digest plasmid pLC1030 (containing the CRISPR guide gRNA to *HIS1* promoter) with KpnI and SacI and transform along with the repair into CaLC3092. NAT resistant colonies were PCR tested for proper integration using oLC237/oLC5733 and oLC238/oLC5735. Verify absence of HisG with oLC1004/oLC238. The *SAP2* promoter was induced to drive expression of the FLP recombinase to excise the NAT marker cassette.

**CaLC5198:** To drive the expression of NAT with *HWP1* promoter in the strain containing *FLO8* at the *HIS1* locus, the NAT-*HIS* cassette was amplified from the genomic DNA of CaLC4920 with oLC3851/oLC3852 and transformed into CaLC5185. HIS prototrophs were PCR tested for proper integration using primers oLC3854/oLC3887 and oLC242/oLC3855.

**CaLC5201:**  To overexpress *TPK2* in the *flo8Δ/flo8Δ* background, the NAT cassette was amplified from pLC605 with primers oLC5697/oLC5696 and transformed into CaLC2897. NAT-resistant transformants were PCR tested for proper integration with primers oLC5698/oLC534 and oLC5699/oLC4714. Absence of *FLO8* alleles was confirmed using oLC5170/oLC2820. The *SAP2* promoter was induced to drive expression of the FLP recombinase to excise the NAT marker cassette.

**CaLC5238/CaLC5239/CaLC5243/CaLC5242/CaLC5237:**

To select for mutants in the *mfg1Δ/mfg1Δ flo8Δ/flo8Δ his1::FLO8/his1::FLO8* background that are able to express the NAT cassette, 4x10^7^ cells of CaLC5198 were plated on YPD+10% serum +250 μg/mL NAT. NAT resistant colonies appeared after 2 days at 37°C. Strains are derived from separate overnights except for CaLC5238 and CaLC5239 which are from the same overnight.

**ScLC5247:** To create a haploid *S. cerevisiae flo8Δ*, ScLC4773 was sporulated in SPO media for 4 days at 30°C with shaking. Spores were treated with 3mg/mL Zymolyase and dissected on rich medium. This spore grew in the absence of *HIS* but required *LEU*. It was able to mate with Mat α cells but not with Mat a cells. Absence of *flo8* was verified with oLC5032/oLC5033.

**ScLC5249:** To create a marker-matched *S. cerevisiae* *flo8Δ* control, ScLC5247 was transformed with pLC155. URA prototrophs were PCR tested with primers oLC1868/oLC1869 to confirm presence of the plasmid.

**ScLC5251:** To complement *S. cerevisiae* *flo8Δ,* ScLC5247 was transformed with pLC987. URA prototrophs were PCR tested with primers oLC4960/oLC4967 to confirm presence of the plasmid.

**CaLC5253:** The *FLO8* knockout construct was PCR-amplified from pLC49 using oLC2818/oLC2819 containing sequence homologous to upstream and downstream regions of *FLO8*, and transformed into CaLC1933. NAT-resistant transformants were PCR tested for proper integration using oLC275/oLC2820 and oLC274/oLC1959. Absence of the wild-type untagged allele was confirmed with oLC1958/oLC1959. The *SAP2* promoter was induced to drive expression of the FLP recombinase to excise the NAT marker cassette.

**CaLC5266:** To overexpress *TPK2* in the *mfg1Δ/mfg1Δ* background, the NAT cassette was amplified from pLC605 with primers oLC5697/oLC5696 and transformed into CaLC1909. NAT-resistant transformants were PCR tested for proper integration with primers oLC5698/oLC534 and oLC5699/oLC4714. The *SAP2* promoter was induced to drive expression of the FLP recombinase to excise the NAT marker cassette.

**CaLC5317:** To complement *FLO8*, pLC1066 containing the wild-type *FLO8* allele was digested with BssHII and transformed into CaLC2897. NAT-resistant transformants were PCR tested for proper integration using oLC921/oLC5170 and oLC274/oLC6371. The *SAP2* promoter was induced to drive expression of the FLP recombinase to excise the NAT marker cassette.

**CaLC5347:** To drive the expression of NAT with *HWP1* promoter in a *flo8Δ/flo8Δ* background, the NAT-*HIS* cassette was amplified from the genomic DNA of CaLC4920 using oLC3851 and oLC3852. The *HWP1p*-NAT-*HIS* construct was transformed into CaLC2897. *HIS* prototrophic transformants were PCR tested for proper upstream integration with oLC2029 and oLC3887 and for downstream integration with oLC242 and oLC3855.

**CaLC5358**: To delete *LRG1* in the *mfg1Δ/mfg1Δ* background, the guide construct containing the CRISPR guide gRNA to *LRG1* was amplified from pLC1073 using oLC5979 and oLC5981. Cas9 was amplified from pLC963 using oLC5974 and oLC5976. The repair construct was amplified from pLC49 using oLC6665 and oLC6666. All three constructs were transformed into CaLC1909. Proper upstream integration was verified using oLC275 and oLC6671. Proper downstream integration was verified using oLC274 and oLC6672. Absence of a wild-type *LRG1* allele was verified using oLC3980 and oLC3981. Absence of a wild-type *MFG1* allele was verified using oLC1920 and oLC1921. The *SAP2* promoter was induced to drive expression of the FLP recombinase to excise the NAT marker cassette.

**CaLC5360:** To delete *LRG1* in the *flo8Δ/flo8Δ* background, the guide construct containing the CRISPR guide gRNA to *LRG1* was amplified from pLC1073 using oLC5979 and oLC5981. Cas9 was amplified from pLC963 using oLC5974 and oLC5976. The repair construct was amplified from pLC49 using oLC6665 and oLC6666. All three constructs were transformed into CaLC2897. Proper upstream integration was verified using oLC275 and oLC6671. Proper downstream integration was verified using oLC274 and oLC6672. Absence of a wild-type *LRG1* allele was verified using oLC3980 and oLC3981. Absence of a wild-type *FLO8* allele was verified using oLC5321 and oLC5506. The *SAP2* promoter was induced to drive expression of the FLP recombinase to excise the NAT marker cassette.

**CaLC5369:** To delete *NRG1*, the guide construct containing the CRISPR guide gRNA to *NRG1* was amplified from pLC1075 using oLC5979 and oLC5981. Cas9 was amplified from pLC963 using oLC5974 and oLC5976. The repair construct was amplified from pLC49 using oLC6663 and oLC6664. All three constructs were transformed into CaLC239. Proper upstream integration was verified using oLC275 and oLC6669. Proper downstream integration was verified using oLC274 and oLC6670. Absence of a wild-type allele was verified using oLC4106 and oLC4107.

**CaLC5380:** To overexpress *IHD1* in the *mfg1Δ/mfg1Δ* background, pLC1077 (containing the CRISPR guide gRNA to the *IHD1* promoter) was digested with KpnI and SacI and transformed into CaLC1909 along with the repair DNA (product PCR-amplified from CaLC3786 gDNA with oLC6647/oLC6733). Upstream integration of the repair was verified with oLC534/oLC6693 and downstream integration was verified with oLC300/oLC6649. Absence of the wild-type promoter was verified using oLC6648/oLC6649. The *SAP2* promoter was induced to drive expression of the FLP recombinase to excise the NAT marker cassette.

**CaLC5383**: To overexpress *TEC1* in the wild-type background, pLC1078 (containing the CRISPR guide gRNA to the *TEC1* promoter) was digested with KpnI and SacI and transformed into CaLC239 along with the repair DNA (product PCR-amplified from CaLC3786 gDNA with oLC6643 and oLC6732). Upstream integration of the repair was verified with oLC534/oLC6644 and downstream integration was verified with oLC300 and oLC6645. Absence of the wild-type promoter was verified using oLC6644 and oLC6645. The *SAP2* promoter was induced to drive expression of the FLP recombinase to excise the NAT marker cassette.

**CaLC5385**: To overexpress *TEC1* in the *mfg1Δ/mfg1Δ* background, pLC1078 (containing the CRISPR guide gRNA to the *TEC1* promoter) was digested with KpnI and SacI and transformed into CaLC1909 along with the repair DNA (product PCR-amplified from CaLC3786 gDNA with oLC6643 and oLC6732. Upstream integration of the repair was verified with oLC534/oLC6644 and downstream integration was verified with oLC300 and oLC6645. Absence of the wild-type *TEC1* promoter was verified using oLC6644 and oLC6645. Absence of the wild-type *MFG1* allele was verified using oLC1920 and oLC1921. The *SAP2* promoter was induced to drive expression of the FLP recombinase to excise the NAT marker cassette.

**CaLC5387:** To overexpress *TEC1* in the *flo8Δ/flo8Δ* background, pLC1078 (containing the CRISPR guide gRNA to the *TEC1* promoter) was digested with KpnI and SacI and transformed into CaLC2897 along with the repair DNA (product PCR-amplified from CaLC3786 gDNA with oLC6643 and oLC6732. Upstream integration of the repair was verified with oLC534 and oLC6644 and downstream integration was verified with oLC300 and oLC6645. Absence of the wild-type *TEC1* promoter was verified using oLC6644 and oLC6645. Absence of the wild-type *FLO8* allele was verified using oLC5321 and oLC5506. The *SAP2* promoter was induced to drive expression of the FLP recombinase to excise the NAT marker cassette.

**CaLC5389:** To delete *NRG1* in the *mfg1Δ/mfg1Δ* background, the guide construct containing the CRISPR guide gRNA to *NRG1* was amplified from pLC1075 using oLC5979 and oLC5981. Cas9 was amplified from pLC963 using oLC5974 and oLC5976. The repair construct was amplified from pLC49 using oLC6663 and oLC6664. All three constructs were transformed into CaLC1909. Proper upstream integration was verified using oLC275 and oLC6669. Proper downstream integration was verified using oLC274 and oLC6670. Absence of a wild-type *NRG1* allele was verified using oLC4106 and oLC4107. Absence of a wild-type *MFG1* allele was verified using oLC1920 and oLC1921.

**CaLC5390:** To delete *NRG1* in the *flo8Δ/flo8Δ* background, the guide construct containing the CRISPR guide gRNA to *NRG1* was amplified from pLC1075 using oLC5979 and oLC5981. Cas9 was amplified from pLC963 using oLC5974 and oLC5976. The repair construct was amplified from pLC49 using oLC6663 and oLC6664. All three constructs were transformed into CaLC2897. Proper upstream integration was verified using oLC275 and oLC6669. Proper downstream integration was verified using oLC274 and oLC6670. Absence of a wild-type *NRG1* allele was verified using oLC4106 and oLC4107. Absence of a wild-type *FLO8* allele was verified using oLC5321 and oLC5506.

**CaLC5450:** To tag Flo8 with a C-terminal TAP tag in a *mfg1Δ/mfg1Δ* background, the TAP construct was amplified from pLC572 with oLC1956 and oLC1957, and transformed into CaLC1909. Proper upstream integration was verified using oLC1634 and oLC1958, and proper downstream integration was verified using oLC1645 and oLC1959.

**CaLC5654:** To tag Flo8 with a C-terminal GFP tag, GFP-NAT was amplified from pLC389 with oLC7026 and oLC7027, and the construct was transformed into CaLC239. Proper upstream integration was verified using oLC5384 and oLC600. Proper downstream integration was verified using oLC1959 and oLC274.

**CaLC5665:** To tag Mfg1 with a C-terminal GFP tag in a background with one allele of *MFG1* deleted, we used transient CRISPR. The sgRNA guide fusion construct was constructed by amplifying Piece A (pLC963 amplified with oLC5978 and oLC7330) and Piece B (pLC963 amplified with oLC5980 and oLC7329), with oLC5981 and oLC5979. Cas9 was amplified from pLC963 with oLC5974 and oLC5976. The GFP-NAT repair construct was amplified from pLC389 with oLC2639 and oLC2640. All three pieces were transformed into CaLC2275. Proper upstream integration was verified with oLC2641 and oLC600, and proper downstream integration was verified with oLC2642 and oLC601. Absence of a wild-type *MFG1* 3’ region was verified using oLC1955 and oLC7187.

**Plasmid construction:**

**pLC984:** To create a *CaMFG1* expression plasmid, *CaMFG1* was amplified from CaLC155 genomic DNA (SC5314) using primers oLC4964/oLC4965, digested with SmaI and BamHI, ligated into pLC155 digested with SmaI and BamHI, and transformed into DH5α cells. Sanger sequenced with oLC5039, oLC883, oLC5038, oLC4964, and oLC4968.

**pLC987:** To create a *CaFLO8* expression vector, *CaFLO8* was amplified from CaLC155 genomic DNA (SC5314) using primers oLC4960/oLC4961, digested with SmaI and BamHI, ligated into pLC155 digested with SmaI and BamHI, and transformed into DH5α cells. Sanger sequenced with oLC883, oLC4960, oLC5036 and oLC5037.

**pLC1008:** To create a vector for CRISPR containing guide RNA targeting *MSS11*, oLC5510/oLC5509 were annealed and ligated into pLC963 digested with BsmBI, and transformed into DH5α cells. Sequencing with oLC4609 verified insertion.

**pLC1030:** To create a vector for CRISPR containing guide RNA targeting the *HIS1* promoter, oLC5678/oLC5679 were annealed and ligated into pLC963 digested with BsmBI, and transformed into DH5α cells. Sequencing with oLC4609 verified insertion.

**pLC1056:** *FLO8* downstream homology was amplified from the genomic DNA of CaLC155 with oLC6369/oLC6370. This product and pLC49 were digested with SacI/SacII, ligated, and transformed into DH5α cells. Sanger sequenced with oLC274.

**pLC1066:** The *FLO8* ORF and upstream homology region was amplified from the genomic DNA of CaLC155 with oLC6367/oLC6368. This product and pLC1056 were digested with ApaI, ligated, and transformed into DH5α cells. Sanger sequenced with oLC274, oLC5170, oLC5321, oLC5036, oLC5322, oLC5037, oLC5384, oLC1958 and oLC275.

**pLC1073:** To create a vector for CRISPR containing guide RNA targeting *LRG1*, oLC6633/oLC6634 were annealed and ligated into pLC963 digested with BsmBI, and transformed into DH5α cells. Sequencing with oLC4609 verified insertion.

**pLC1075:** To create a vector for CRISPR containing guide RNA targeting *NRG1*, oLC6637/oLC6638 were annealed and ligated into pLC963 digested with BsmBI, and transformed into DH5α cells. Sequencing with oLC4609 verified insertion.

**pLC1077:** To create a vector for CRISPR containing guide RNA targeting the *IHD1* promoter, oLC6691/oLC6692 were annealed and ligated into pLC963 digested with BsmBI, and transformed into DH5α cells. Sequencing with oLC4609 verified insertion.

**pLC1078:** To create a vector for CRISPR containing guide RNA targeting the *TEC1* promoter, oLC6689/oLC6690 were annealed and ligated into pLC963 digested with BsmBI, and transformed into DH5α cells. Sequencing with oLC4609 verified insertion.

**Supplemental References:**

1. Lavoie H, Sellam A, Askew C, Nantel A, Whiteway M (2008) A toolbox for epitope-tagging and genome-wide location analysis in *Candida albicans*. BMC Genomics 9: 578.

2. Noble SM, Johnson AD (2005) Strains and strategies for large-scale gene deletion studies of the diploid human fungal pathogen *Candida albicans*. Eukaryot Cell 4: 298-309.

3. Mumberg D, Muller R, Funk M (1995) Yeast vectors for the controlled expression of heterologous proteins in different genetic backgrounds. Gene 156: 119-122.

4. Veri AO, Miao Z, Shapiro RS, Tebbji F, O'Meara TR, et al. (2018) Tuning Hsf1 levels drives distinct fungal morphogenetic programs with depletion impairing Hsp90 function and overexpression expanding the target space. PLoS Genet 14: e1007270.

5. Jones T, Federspiel NA, Chibana H, Dungan J, Kalman S, et al. (2004) The diploid genome sequence of *Candida albicans*. Proc Natl Acad Sci U S A 101: 7329-7334.

6. Shen J, Guo W, Kohler JR (2005) *CaNAT1*, a heterologous dominant selectable marker for transformation of *Candida albicans* and other pathogenic *Candida* species. Infect Immun 73: 1239-1242.

7. Mumberg D, Muller R, Funk M (1995) Yeast vectors for the controlled expression of heterologous proteins in different genetic backgrounds. Gene 156: 119-122.

8. Gerami-Nejad M, Berman J, Gale CA (2001) Cassettes for PCR-mediated construction of green, yellow, and cyan fluorescent protein fusions in *Candida albicans*. Yeast 18: 859-864.
