# Supplementary figures and images for: Functional divergence of a global regulatory complex governing fungal filamentation
Source: PLoS Genet. 2019 Jan 7;15(1):e1007901. doi: 10.1371/journal.pgen.1007901 (PMC6336345; doi:10.1371/journal.pgen.1007901)

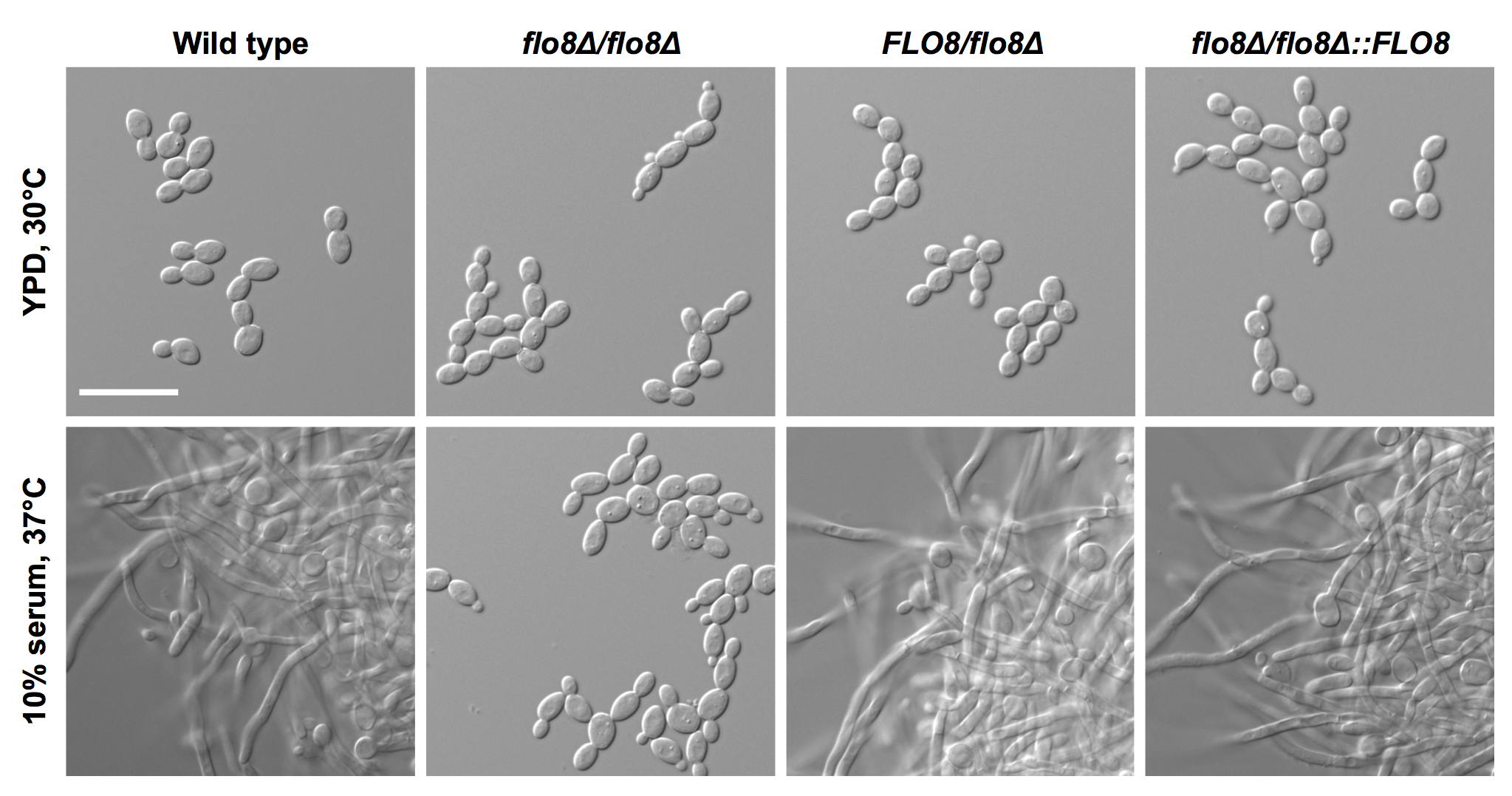

Supplement: S1 Fig — Cells were grown in the conditions indicated and imaged after 3.5 hours. Scale bar is 20 μm. (TIFF) [file pgen.1007901.s002.tiff]

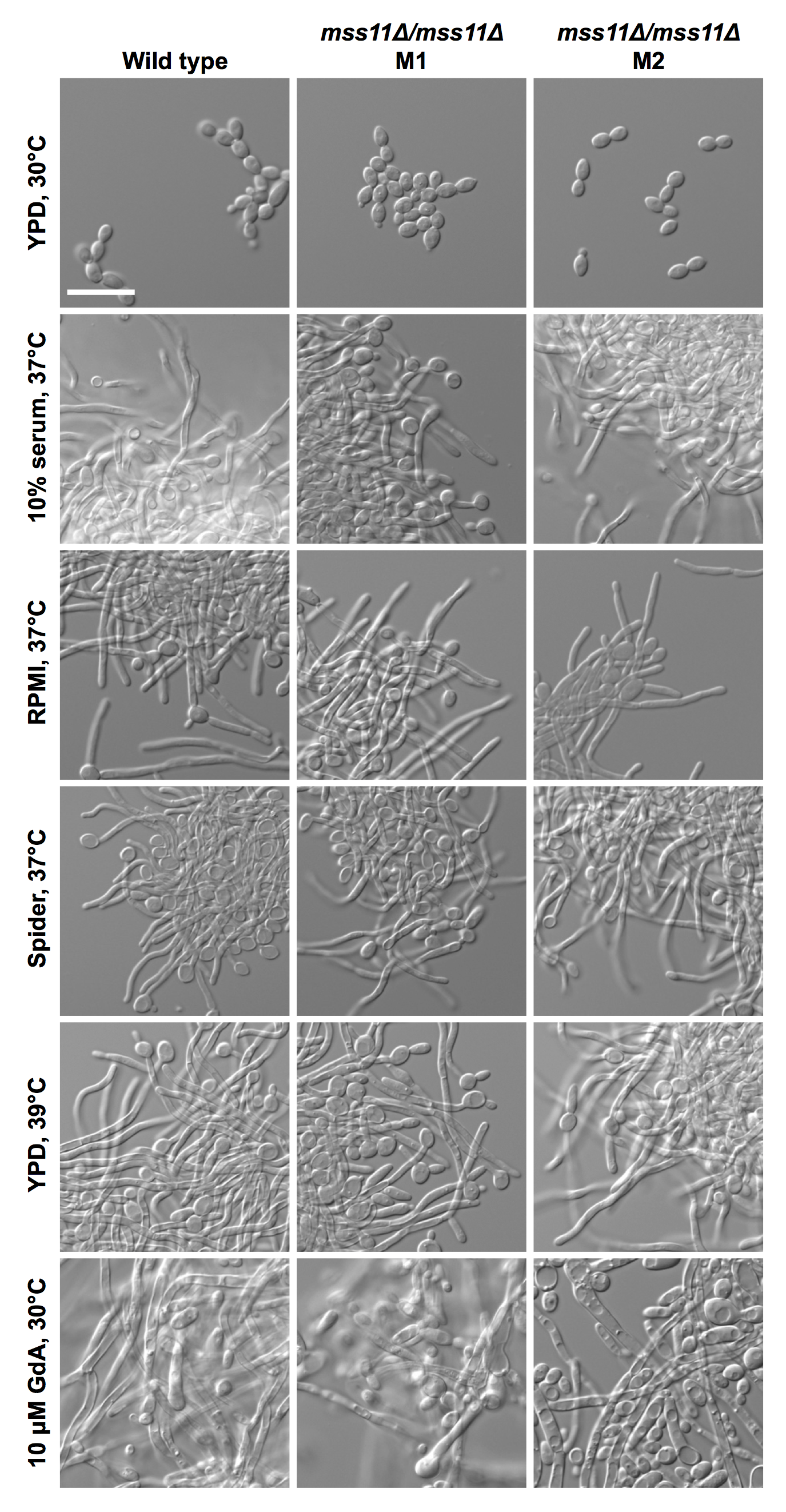

Supplement: S2 Fig — Two independently generated mss11Δ/mss11Δ mutants, M1 from Fig 1 and a second mutant M2, show no defect in filamentation in response to any cue tested. Cells were grown in the conditions indicated, and were imaged after 3.5 hours, except for those grown in the presence of GdA, which were imaged after 24 hours. Scale bar is 20 μm. (TIFF) [file pgen.1007901.s003.tiff]

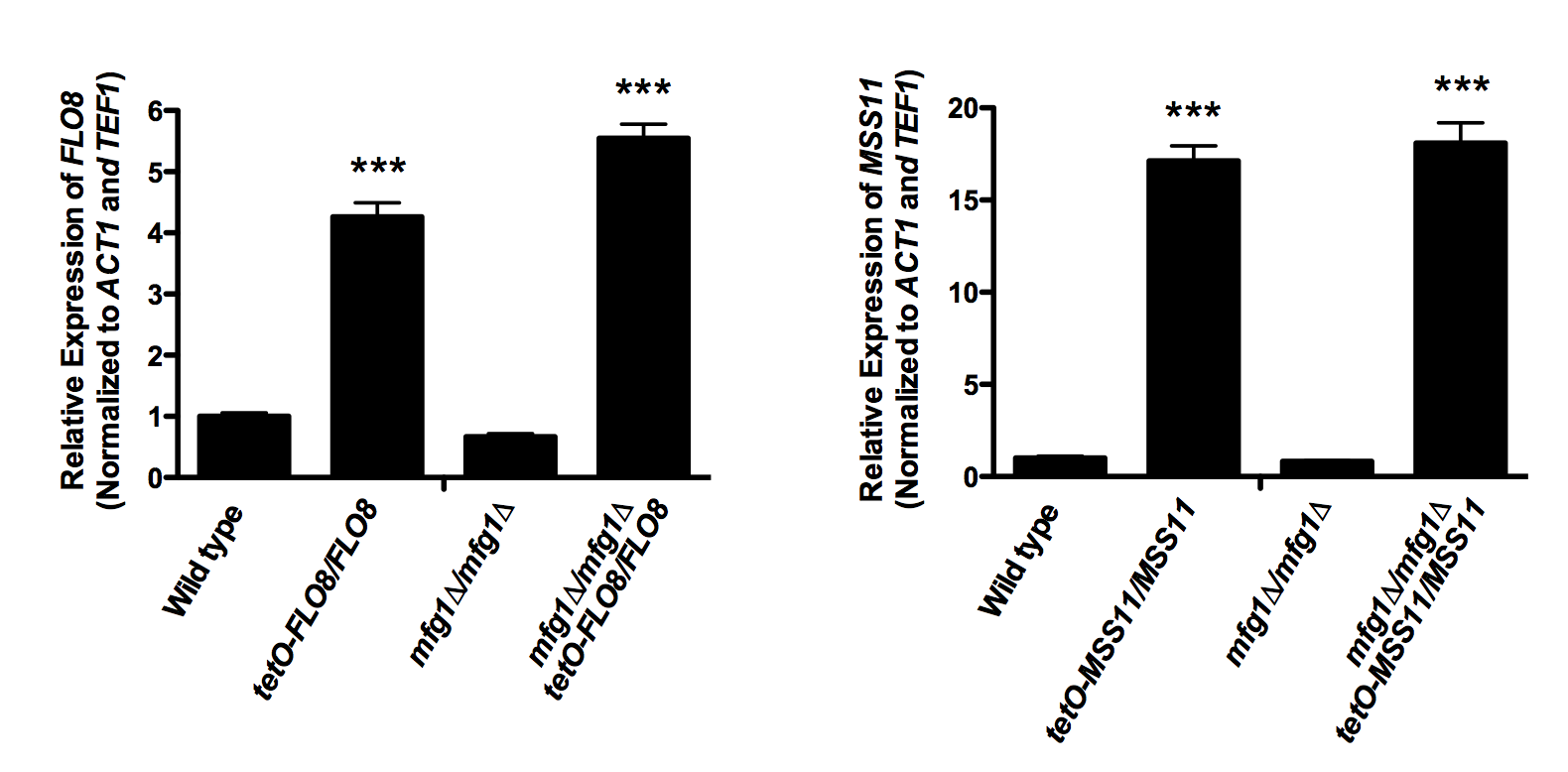

Supplement: S3 Fig — Quantification of overexpression of FLO8 or MSS11 in the mfg1Δ/mfg1Δ mutant by qRT-PCR. Cells were grown in YPD at 30°C for 3 hours. Transcript levels were normalized to ACT1 and TEF1 and error bars represent standard error of technical triplicates. Assays were performed in biological duplicate. Asterisks indicate P< 0.0001 (***) relative to parental strain (two-tailed unpaired t-test). (TIFF) [file pgen.1007901.s004.tiff]

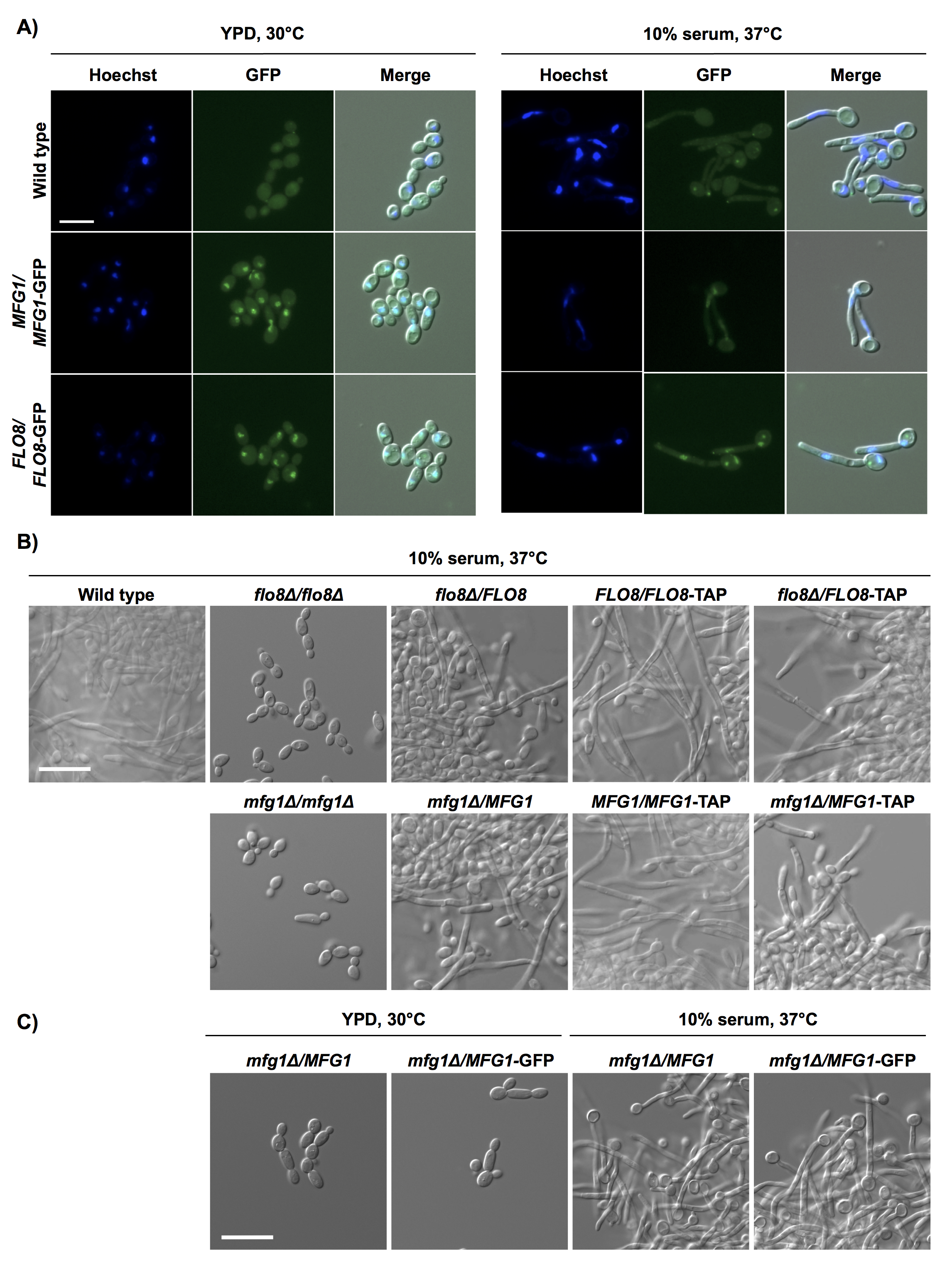

Supplement: S4 Fig — A) Cells expressing Mfg1-GFP or Flo8-GFP were grown in either YPD at 30°C or YPD with 10% serum at 37°C for 1 hour. Cells were treated with Hoechst 33342 dye to monitor nuclear localization. B) Cells expressing Flo8-TAP or Mfg1-TAP were grown in YPD with 10% serum at 37°C for 6 hours. Scale bar is 20 μm. C) Mfg1-GFP cells were grown in YPD at 30°C or in YPD with 10% serum at 37°C for 2 hours. Scale bar is 20 μm. (TIFF) [file pgen.1007901.s005.tiff]

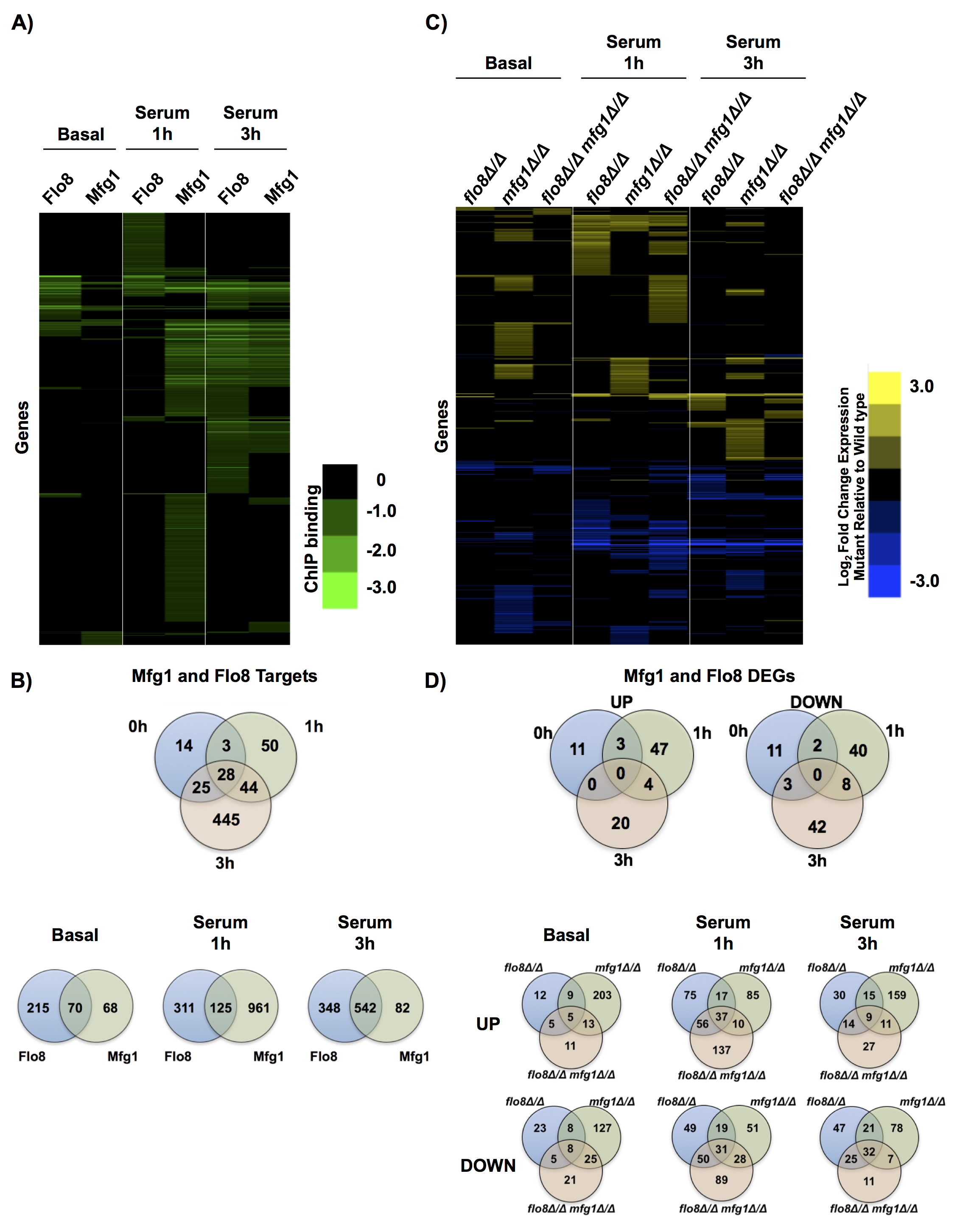

Supplement: S5 Fig — A) Cells for ChIP-chip were grown in YPD at 30°C until mid-log phase (basal), and then treated for 1 hour or 3 hours in 10% serum at 37°C. Genes were clustered using Cluster Gene 3.0 and visualized using JavaTreeView, where green represents an increased binding intensity. B) ChIP-chip analysis reveals a core set of genes bound by both Flo8 and Mfg1 under basal (untreated 30°C) and filament-inducing (serum 37°C) conditions (top Venn diagram). Our analysis also identified many unique targets under all environmental conditions, as well as substantial changes in promoter binding upon exposure to serum (bottom Venn diagrams). C) Wild-type, flo8Δ/flo8Δ, mfg1Δ/mfg1Δ, or flo8Δ/flo8Δ mfg1Δ/mfg1Δ strains were grown as in A) for transcriptional analysis by microarray. Heat map was generated as in A. Plotted are the log2 fold-change in expression of the mutant strain relative to wild type. D) Microarray analysis reveals that Flo8 and Mfg1 have distinct effects on gene expression (DEG: differentially expressed gene) and identifies temporal transcriptional changes that occur in response to serum. (TIFF) [file pgen.1007901.s006.tiff]

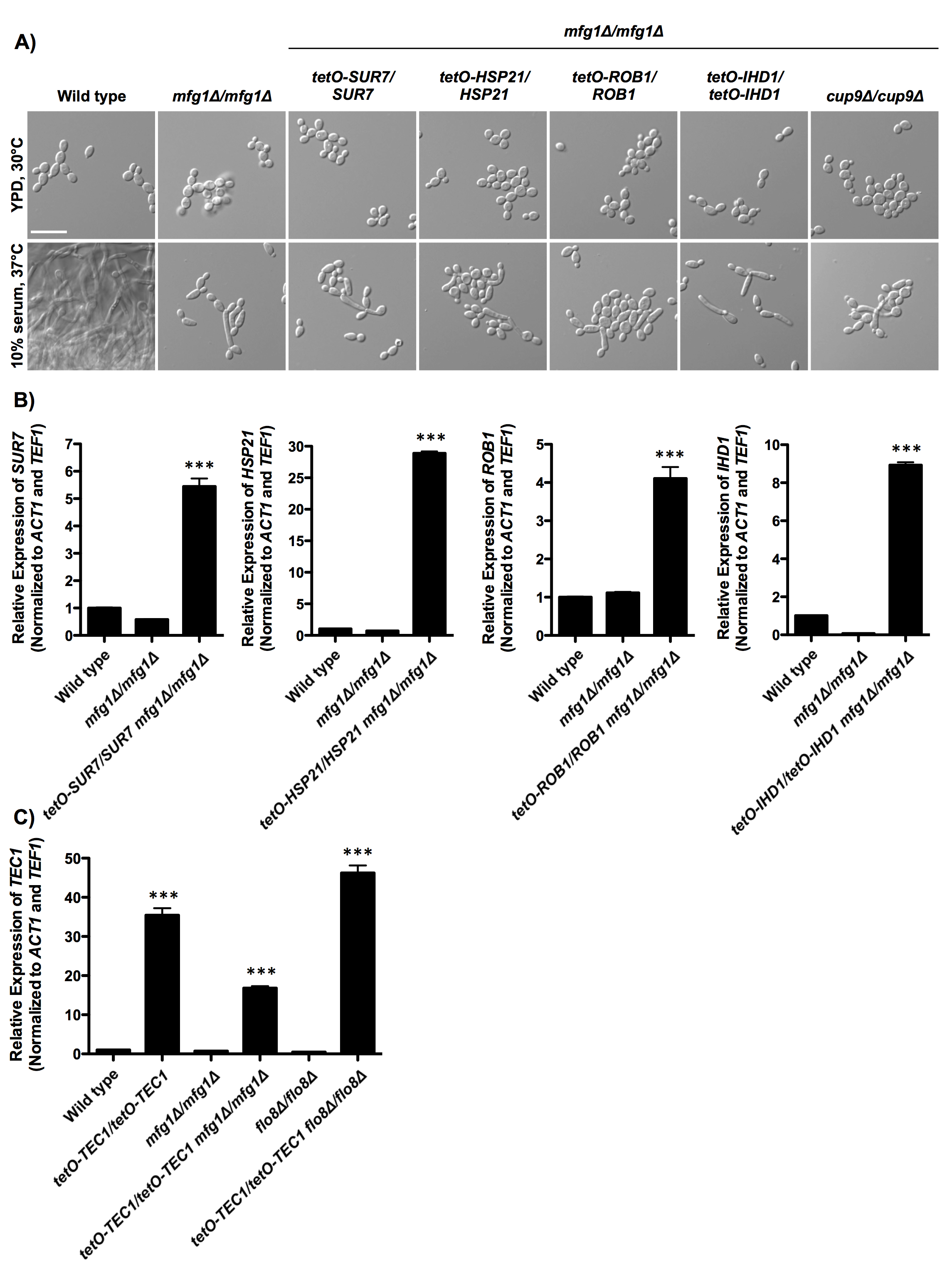

Supplement: S6 Fig — A) Select genes both bound and transcriptionally regulated by Mfg1 upon exposure to serum were overexpressed (positive regulators of filamentation), or deleted (negative regulator of filamentation) in an mfg1Δ/mfg1Δ mutant. Cells were grown in YPD at 30°C, or in YPD with 10% serum at 37°C for 5 hours. Scale bar is 20 μm. B) Quantification of overexpression of target genes by qRT-PCR. Overexpression of each target open reading frame was achieved by replacing the native promoter of one or both alleles for each gene with a tetracycline-repressible promoter, tetO. Cells were grown in YPD in the presence of 10% serum at 37°C for 6 hours. Transcript levels were monitored using qRT-PCR and normalized to ACT1 and TEF1. Error bars represent standard error of technical triplicates. Assays were performed in biological duplicate. Asterisks indicate P < 0.0001 (***), relative to the parental strain (one-way ANOVA, Bonferroni Multiple Comparison Test). C) Replacing both native TEC1 promoters with the strong tetO promoter results in increased TEC1 expression. Cells were grown in YPD at 30°C for 4 hours. Transcript levels were monitored using qRT-PCR and normalized to ACT1 and TEF1. Error bars represent standard error of technical triplicates. Assays were performed in biological duplicate. Asterisks indicate P < 0.0001 (***), relative to the parental strain (one-way ANOVA, Bonferroni Multiple Comparison Test). (TIFF) [file pgen.1007901.s007.tiff]

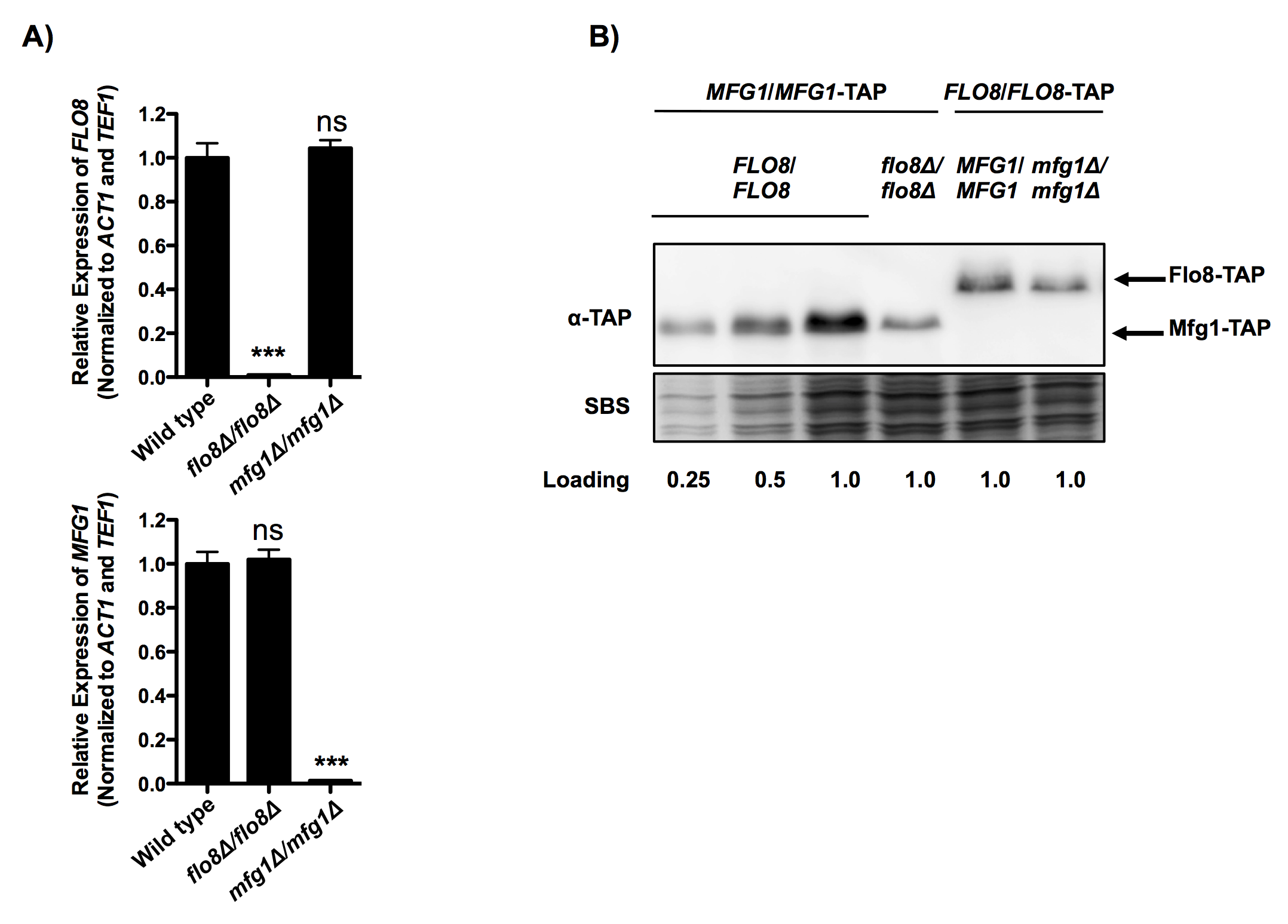

Supplement: S7 Fig — A) Deletion of FLO8 or MFG1 does not alter the expression of the other regulator. Cells were grown in YPD at 30°C for 4 hours. Transcript levels were monitored using qRT-PCR and normalized to ACT1 and TEF1. Error bars represent standard error of technical triplicates. Assays were performed in biological duplicate. Asterisks indicate P < 0.0001 (***), relative to the wild type (two-tailed unpaired t-test). B) Protein levels of Mfg1-TAP and Flo8-TAP in wild-type cells or cells lacking the other regulator were monitored with immunoblotting. Cells were grown in YPD at 30°C for 5 hours. Staining with Simple Blue Safe (SBS) was used as a loading control. ‘Loading’ indicates relative amount of lysate loaded. (TIFF) [file pgen.1007901.s008.tiff]

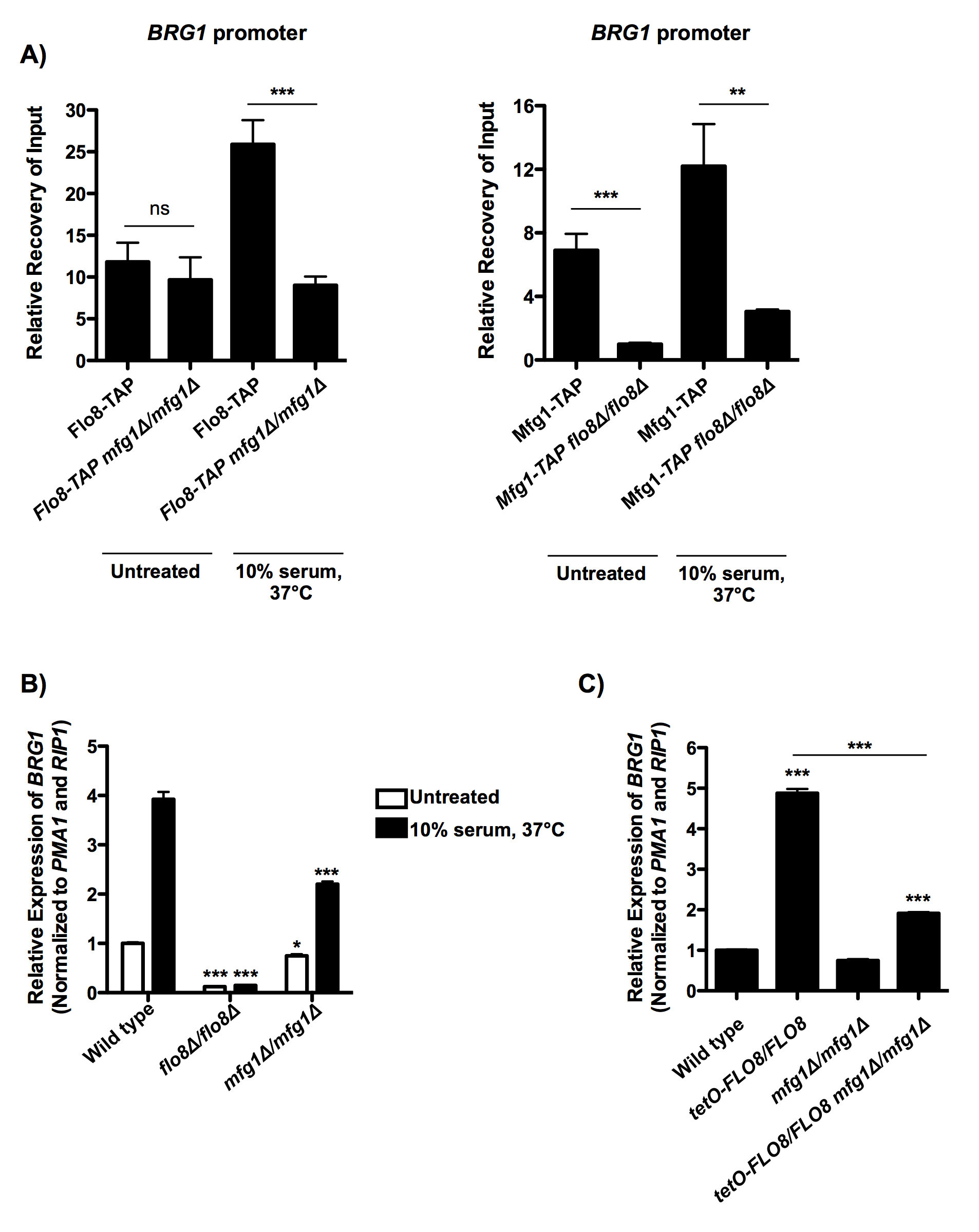

Supplement: S8 Fig — A) Binding of Flo8-TAP and Mfg1-TAP to the promoter of BRG1 was assessed using ChIP-qPCR. Shown is the fold-enrichment over the untagged parental strain, which is set at 1. Asterisks indicate P < 0.01 (**) or P < 0.001 (***), relative to the wild-type parent in the respective condition (two-tailed unpaired t-test). Error bars represent the standard deviation of technical triplicates. Assays were performed in biological duplicate. B) BRG1 expression is decreased in the absence of Flo8 or Mfg1. Cells were grown in YPD at 30°C for 3.5 hours, and then transferred to 37°C YPD with 10% serum for 1 hour. Transcript levels were monitored using qRT-PCR and normalized to PMA1 and RIP1. Error bars represent standard error of technical triplicates. Assays were performed in biological duplicate. Asterisks indicate P < 0.001 (***) or P < 0.05 (*), relative to the wild-type strain in each respective condition (two-way ANOVA, Bonferroni Multiple Comparison Test). C) BRG1 expression is increased in strains overexpressing FLO8. Cells were grown in YPD at 30°C for 4.5 hours. Transcript levels were monitored using qRT-PCR and normalized to PMA1 and RIP1. Error bars represent standard error of technical triplicates. Assays were performed in biological duplicate. Asterisks indicate P < 0.0001 (***), relative to the respective parental strain, or as indicated (one-way ANOVA, Bonferroni Multiple Comparison Test). (TIFF) [file pgen.1007901.s009.tiff]

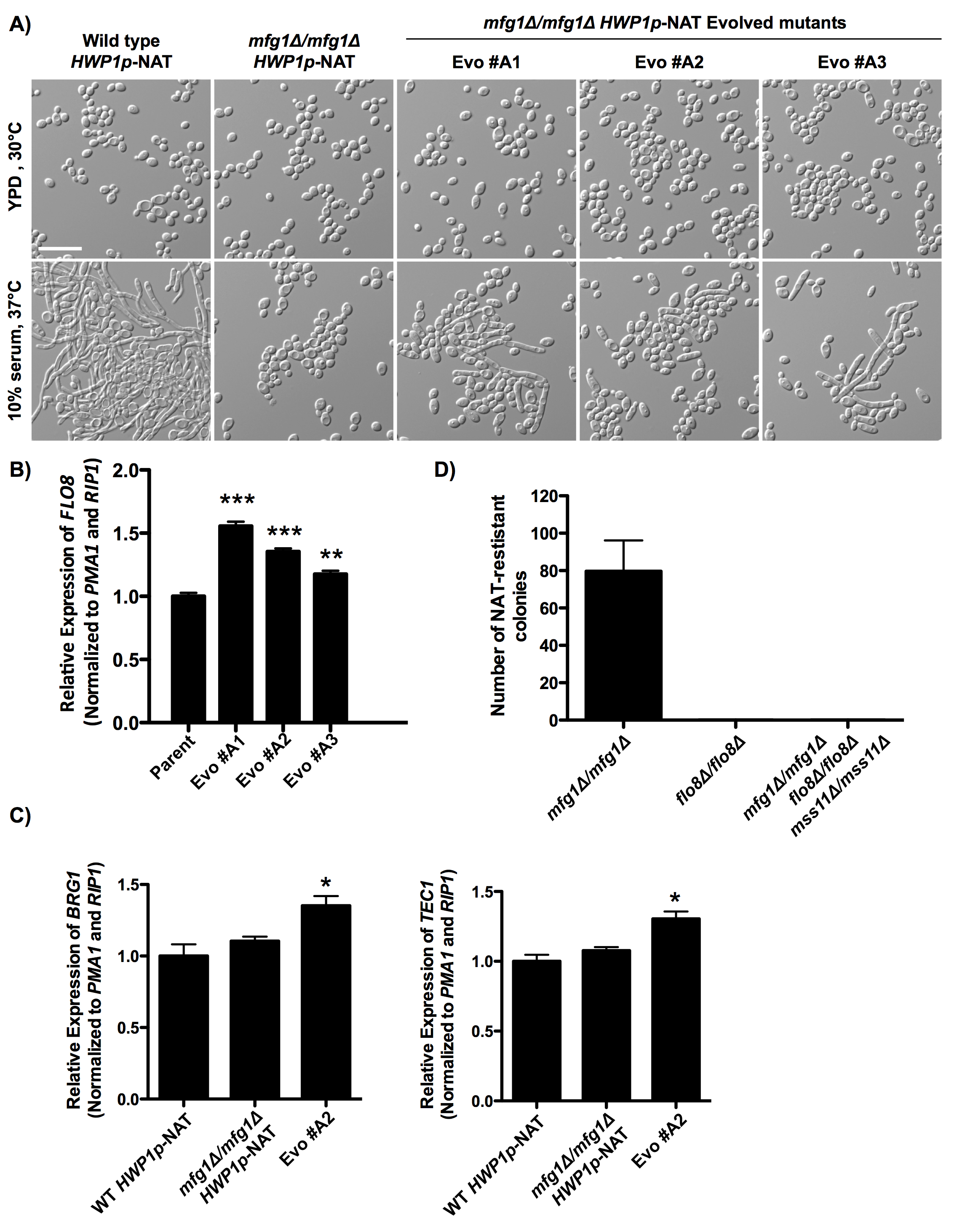

Supplement: S9 Fig — A) Evolved mutants revert to yeast form after 24 hours of growth in serum. Cells were grown in YPD at 30°C or in YPD with 10% serum at 37°C for 24 hours. Scale bar represents 20 μm. B) Expression of FLO8 is increased in evolved lineages with increased FLO8 copy number. Cells were grown in YPD at 30°C for 3.5 hours. Transcript levels were monitored using qRT-PCR and normalized to PMA1 and RIP1. Error bars represent standard error of technical triplicates. Assays were performed in biological duplicate. Asterisks indicate P < 0.001 (***) and P < 0.01 (**), relative to the parent (two-tailed unpaired t-test). C) Expression of BRG1 and TEC1 are increased in the evolved lineage Evo #A2 in the presence of 10% serum at 37°C. Cells were grown in YPD at 30°C for 3.5 hours, before being transferred to 37°C YPD with 10% serum for 1 hour. Transcript levels were monitored using qRT-PCR and normalized to PMA1 and RIP1. Error bars represent standard error of technical triplicates. Assays were performed in biological duplicate. Asterisks indicate P < 0.05 (*), relative to the parental strain (two-tailed unpaired t-test). D) NAT-resistant colonies were not obtained when the flo8Δ/flo8Δ mutant or the mfg1Δ/mfg1Δ flo8Δ/flo8Δ mss11Δ/mss11Δ triple mutant were plated on 10% serum and 250 μg/mL of NAT. 4 x10^7^ cells were plated in triplicate, and incubated for 72 hours. (TIFF) [file pgen.1007901.s010.tiff]

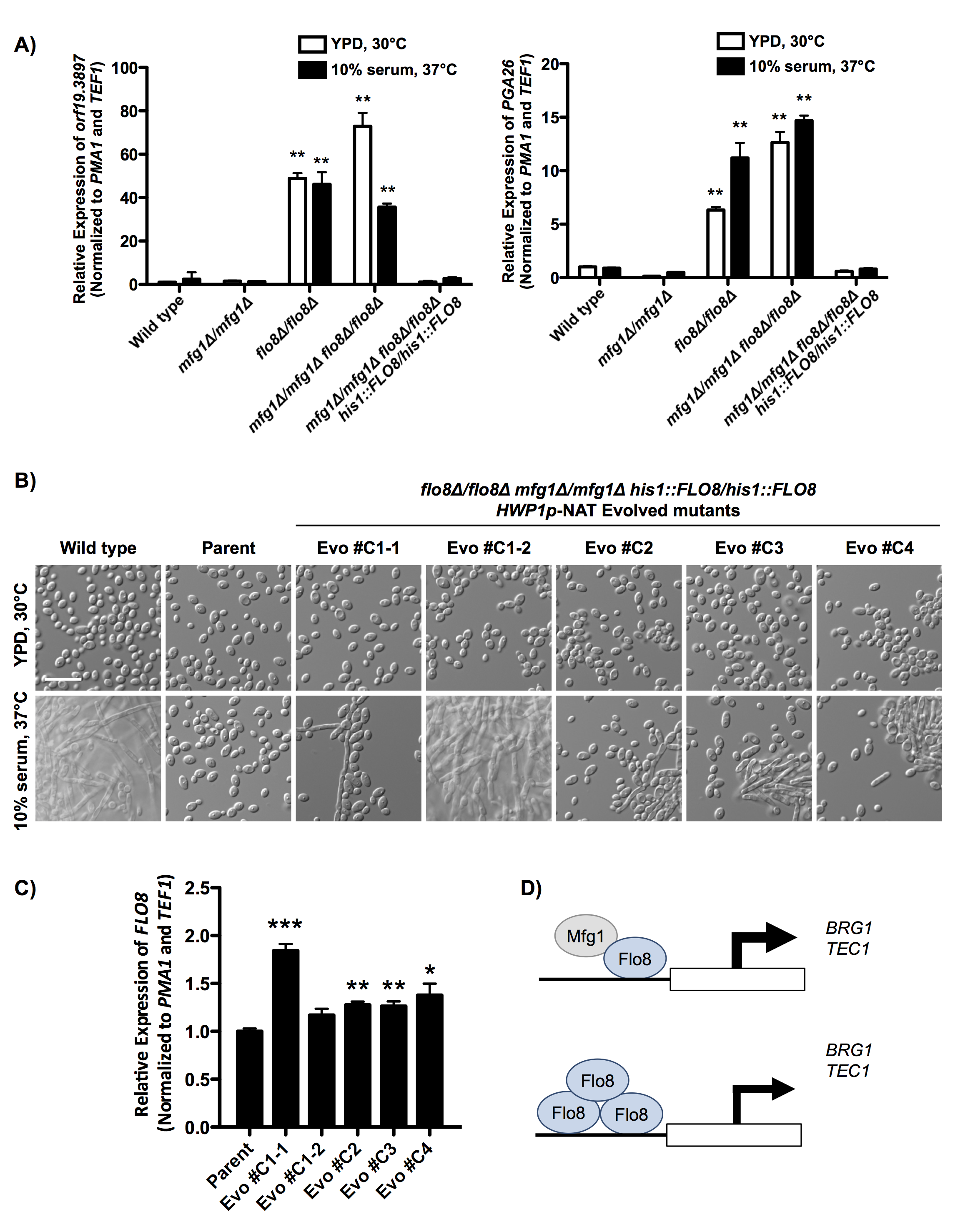

Supplement: S10 Fig — A) Expression of Flo8-repressed genes does not increase in the strain in which FLO8 is at the HIS1 locus on chromosome 5. Cells were grown in YPD at 30°C for 4 hours or in YPD with 10% serum at 37°C for 3 hours. Transcript levels were monitored using qRT-PCR and normalized to PMA1 and TEF1. Error bars represent standard error of technical triplicates. Assays were performed in biological duplicate. Asterisks indicate P < 0.001 (**), relative to the wild type (two-way ANOVA, Bonferroni correction). B) Most filament-capable mfg1Δ/mfg1Δ mutants with FLO8 at a non-native locus revert to yeast form growth after 24 hours in serum. Cells were grown in YPD at 30°C or in YPD with 10% serum at 37°C for 24 hours. Mutants Evo #C1-1 and Evo #C1-2 arose from the same overnight culture but showed distinct phenotypes in terms of their ability to filament at a later time point. Mutants Evo #C1, C2, C3, and C4 are all independently generated. Scale bar is 20 μm. C) Expression of FLO8 is most increased in the evolved lineage with increased FLO8 copy number. Cells were grown in YPD at 30°C for 3.5 hours. Transcript levels were monitored using qRT-PCR and normalized to PMA1 and TEF1. Error bars represent standard error of technical triplicates. Assays were performed in biological duplicate. Asterisks indicate P < 0.001 (***), P < 0.01 (**), or P < 0.05 (*) relative to the parent (two-tailed unpaired t-test). D) A model depicting the roles of Mfg1 and Flo8 in governing expression of key regulators of filamentation. Mfg1 and Flo8 are both required for optimal binding to the promoters of BRG1 and TEC1, however, overexpression of FLO8 in the absence of Mfg1 is sufficient to drive filamentation and expression of BRG1 and TEC1. (TIFF) [file pgen.1007901.s011.tiff]
